# Could Circumcision of HIV-Positive Males Benefit Voluntary Medical Male Circumcision Programs in Africa? Mathematical Modeling Analysis

**DOI:** 10.1371/journal.pone.0170641

**Published:** 2017-01-24

**Authors:** Susanne F. Awad, Sema K. Sgaier, Fiona K. Lau, Yousra A. Mohamoud, Bushimbwa C. Tambatamba, Katharine E. Kripke, Anne G. Thomas, Naomi Bock, Jason B. Reed, Emmanuel Njeuhmeli, Laith J. Abu-Raddad

**Affiliations:** 1 Infectious Disease Epidemiology Group, Weill Cornell Medical College in Qatar, Cornell University, Qatar Foundation, Education City, Doha, Qatar; 2 Surgo Foundation, Washington, District of Columbia, United States of America; 3 Department of Global Health and Population, Harvard T. H. Chan School of Public Health, Boston, Massachusetts, United States of America; 4 Department of Global Health, University of Washington, Seattle, Washington, United States of America; 5 Ministry of Community Development and Mother and Child Health, Lusaka, Zambia; 6 Health Policy Initiative, Avenir Health, Washington, District of Columbia, United States of America; 7 Naval Health Research Center, U.S. Department of Defense, San Diego, California, United States of America; 8 Division of Global HIV/AIDS, Center for Global Health, Centers for Disease Control and Prevention, Atlanta, Georgia, United States of America; 9 Jhpiego, Washington, District of Columbia, United States of America; 10 United States Agency for International Development, Washington, District of Columbia, United States of America; 11 Department of Healthcare Policy and Research, Weill Cornell Medical College, Cornell University, New York, New York, United States of America; 12 College of Public Health, Hamad bin Khalifa University, Qatar Foundation, Education City, Doha, Qatar; University of Washington, UNITED STATES

## Abstract

**Background:**

The epidemiological and programmatic implications of inclusivity of HIV-positive males in voluntary medical male circumcision (VMMC) programs are uncertain. We modeled these implications using Zambia as an illustrative example.

**Methods and Findings:**

We used the Age-Structured Mathematical (ASM) model to evaluate, over an intermediate horizon (2010–2025), the effectiveness (number of VMMCs needed to avert one HIV infection) of VMMC scale-up scenarios with varying proportions of HIV-positive males. The model was calibrated by fitting to HIV prevalence time trend data from 1990 to 2014. We assumed that inclusivity of HIV positive males may benefit VMMC programs by increasing VMMC uptake among higher risk males, or by circumcision reducing HIV male-to-female transmission risk. All analyses were generated assuming no further antiretroviral therapy (ART) scale-up.

The number of VMMCs needed to avert one HIV infection was projected to increase from 12.2 VMMCs per HIV infection averted, in a program that circumcises only HIV-negative males, to 14.0, in a program that includes HIV-positive males. The proportion of HIV-positive males was based on their representation in the population (e.g. 12.6% of those circumcised in 2010 would be HIV-positive based on HIV prevalence among males of 12.6% in 2010). However, if a program that only reaches out to HIV-negative males is associated with 20% lower uptake among higher-risk males, the effectiveness would be 13.2 VMMCs per infection averted. If improved inclusivity of HIV-positive males is associated with 20% higher uptake among higher-risk males, the effectiveness would be 12.4. As the assumed VMMC efficacy against male-to-female HIV transmission was increased from 0% to 20% and 46%, the effectiveness of circumcising regardless of HIV status improved from 14.0 to 11.5 and 9.1, respectively. The reduction in the HIV incidence rate among females increased accordingly, from 24.7% to 34.8% and 50.4%, respectively.

**Conclusion:**

Improving inclusivity of males in VMMC programs regardless of HIV status increases VMMC effectiveness, if there is moderate increase in VMMC uptake among higher-risk males and/or if there is moderate efficacy for VMMC against male-to-female transmission. In these circumstances, VMMC programs can reduce the HIV incidence rate in males by nearly as much as expected by some ART programs, and additionally, females can benefit from the intervention nearly as much as males.

## Introduction

Randomized controlled trials (RCT) reported approximately 60% efficacy for male circumcision against heterosexual HIV acquisition among males [1–3]. Current evidence suggests that voluntary male medical circumcision (VMMC) is an effective, cost-effective, and cost-saving HIV prevention intervention [4–10]. The World Health Organization (WHO) and the Joint United Nations Programme on HIV/AIDS (UNAIDS) recommend VMMC as part of a comprehensive HIV prevention package in several countries in East and Southern Africa [11, 12].

WHO and UNAIDS recommend that HIV testing and counseling (HTC) be offered to all clients presenting for VMMC, as part of the comprehensive package of HIV prevention services [11]. While VMMC for HIV prevention purposes is not recommended for uncircumcised HIV-positive males, WHO and UNAIDS stipulate that HIV positive individuals requesting VMMC should not be denied the service solely due to HIV status, to avoid stigma [11]. To date, HTC acceptance rates have generally been high [11, 13]. This is a positive outcome given that awareness of HIV status is an important component of HIV prevention [13, 14], and that HIV-positive males are linked to HIV care and treatment services [15]. At the same time, though, qualitative analyses suggested that there is a risk that higher-risk HIV-negative males might forgo VMMC if they perceive that HTC is mandatory, for fear of an HIV-positive diagnosis or that they will be identified as HIV-positive by their communities [16, 17].

A factor for policymakers to consider when determining whether VMMC recruitment activities should include all males, regardless of HIV status/awareness, is the potential efficacy of male circumcision against HIV transmission from a circumcised HIV-positive male to his female sexual partners (*male-to-female HIV transmission*). The evidence is mixed on this efficacy [18–25]. Females with circumcised partners have been found to have an equal [18, 22, 26], lower [21, 23, 27–29], or higher [18, 30, 31] risk of acquiring HIV than those with uncircumcised partners. Two studies attempted to pool existing evidence on this effect [19, 20]. The first study, a systematic review and meta-analysis in 2009, reported that male circumcision had a 20% efficacy against male-to-female HIV transmission, but with no statistical significance [19]. The second study, a meta-analysis in 2010, pooled the outcome of two quality measures from two observational cohorts and reported a statistically significant 46% efficacy for male circumcision [20].

The only RCT to directly test for a difference in HIV transmission risk to females based on males’ circumcision status was stopped early, for futility [18]. The study demonstrated a statistically insignificant increased risk of infection in females who resumed sex before the circumcision wounds of their partners had healed, in comparison with the HIV transmission risk of females with uncircumcised partners and of females who did not resume sex before their partners circumcision wounds had healed [18]. There is evidence that 4% to 31% of males—including those identified as HIV-positive prior to VMMC—resume sex before their wounds heal [25, 31–34]. If more than 30% of males resume sexual activity too soon, one model of the impact suggests that there could be a net negative effect on their female partners within the first year post-circumcision: that is, the intervention would generate more new infections among females [34]. This model, however, only assessed the impact of VMMC on the HIV epidemic over one year and did not capture longer term impact. The model’s finding was also generated assuming high relative risk of male-to-female HIV transmission during wound healing and a consistent coital frequency [34]. These two assumptions may be unrealistic, and lead to an underestimation of the beneficial impact of VMMC and an overestimation of the negative impact on females. Regardless, this modeling study and other studies show that even in the first year, early resumption of sex will not offset the overall epidemiological benefits of VMMC programs [7, 8, 10, 20, 25, 34].

In this study, we used mathematical modeling to assess the epidemiological and some programmatic implications of including HIV-positive males in VMMC programs. The primary outcome of interest was the *effectiveness* of VMMC as an HIV intervention, defined as the number of VMMCs needed per HIV infection averted. The lower the number of VMMCs required to avert one infection, the better the effectiveness of VMMC in that scenario. The secondary outcome of interest was the *scale of reduction in the risk of HIV exposure*, defined as HIV incidence rate reduction. This measure was calculated by comparing the population-level HIV incidence rate in the presence of the VMMC scale-up program with a counter-factual scenario with no VMMC scale-up program.

The intervention’s impact on the HIV epidemic was evaluated in two time horizons: an intermediate-term time horizon (2010–2025), and a long-term time horizon (2010–2035). We mainly focused in our analyses on the intermediate time horizon, as the goal of the VMMC program is to impact the HIV epidemic in the near to intermediate future [35].

Our analysis focused on Zambia as an illustrative example, with the purpose of assessing the implications of including HIV-positive males in VMMC programs in countries with high HIV prevalence and low circumcision rates. The VMMC program in Zambia aims to reduce HIV incidence by circumcising 80% of 15–49 year old males between 2010 and 2017, for a total of 1.95 million circumcisions [36]. While Zambia’s VMMC program does not exclude HIV-positive males who meet the other eligibility requirements, about 99% of VMMC clients to date have been HIV-negative males [37].

## Methods

### Modeling Approach

We used the Age-Structured Mathematical (ASM) model [38, 39] to address the research questions of this study. Detailed description of the ASM model and parameterization, and description of the HIV epidemic in Zambia, can be found in S1 Text and Awad et al. [38]. Briefly, the ASM model is a population-level deterministic compartmental mathematical model that describes the heterosexual transmission of HIV in a given population (S1 Fig). Deterministic modelling is an appropriate choice to examine HIV dynamics and the effectiveness of intervention programs at the national level for such large HIV epidemics. The model consists of a set of coupled nonlinear differential equations that stratify the population into compartments according to sex (males and females), circumcision status of males, age group, sexual risk group, HIV status, and stage of HIV infection. Progression of HIV infection is divided into three stages: acute, chronic, and advanced.

The model disaggregates the population into 20 age groups with each group representing a five-year age band (0–4, 5–9, …, 95–99 year old). To account for heterogeneity in sexual risk behavior, we incorporated six sexual risk groups in the population with increasing level of sexual risk behavior [38]. The distribution of the population across the six risk groups was informed by empirical data from sub-Saharan Africa (SSA) on the distribution of the number of sexual partners over the past year [40–44]. Accordingly, the risk group in the model representing the general population (risk group 1) has the lowest risk of acquiring HIV in the study population. The risk group representing key populations to the HIV epidemic such as female sex workers and their male clients (risk group 6) has the highest risk of acquiring HIV [38]. The overall level of sexual risk behavior was assumed to vary during the HIV epidemic, as evidence has shown substantial reductions in the risk of HIV exposure across SSA [45]. Further details on sexual risk behavior parameterization and its time evolution can be found in S1 Text. Individuals of different risk and age groups interact according to mixing matrices that describe sexual networking and incorporate both an assortative component (choosing partners from within their risk or age group) and proportionate component (choosing partners with no preferential bias based on the type of risk group or age group) mixing [38, 46, 47].

A proportion of males in the population of Zambia was assumed to be circumcised through traditional circumcision, as per Demographic and Health Surveys data [48]. The efficacy of VMMC against HIV acquisition among males was modeled as a proportional reduction in the risk of HIV acquisition among circumcised males. The efficacy of VMMC against male-to-female HIV transmission was modeled as a fractional reduction in the transmission probability per coital act. Existing data and the postulated biological mechanisms of action do not suggest differential effect of VMMC by HIV stage of infection (i.e. acute, chronic and advanced stages) [19, 20]. Accordingly, we assumed that each of the efficacies of VMMC against HIV acquisition among males and against male-to-female HIV transmission is not stage dependent and equal for all HIV stages. The model accommodates the possibility of risk compensation among circumcised males following VMMC, but we assumed 0% risk compensation in our predictions based on inconclusive empirical evidence to support increases in risk behavior [49–53].

### Data Sources and Model Fitting

The model was parameterized using current epidemiological and natural history data from SSA [38], as well as through model fitting for some of the parameters. HIV biological and behavioral parameter values are described in Awad et al. [38]. The country-specific time series of HIV prevalence data was obtained from UNAIDS estimates between 1990 and 2011 [54]. Baseline male circumcision prevalence, reflecting background non-VMMC program circumcisions in Zambia, was obtained from the Zambia Demographic and Health Survey 2007 [55]. Demographics such as total population size and future projections were obtained from the database of the Population Division of the United Nations Department of Economic and Social Affairs [56].

The model was fitted to HIV prevalence time series data in the years between 1990 and 2014, using a nonlinear least-square fitting method. The technique was implemented in MATLAB version 2015a [57] using the Nelder-Mead simplex algorithm (*fminsearch*) as described in Lagarias et al. [58].

### VMMC Intervention Scenarios

In the modeled intervention scenarios, the scale-up of the VMMC program was assumed to start in 2010, with a “catch-up” phase lasting seven years (to 2017) and a “sustainability” phase lasting another eight years (to 2025). The catch-up phase focused on the 15–49 year old population and ends by achieving 80% VMMC coverage in this age bracket. In the sustainability phase, the attained coverage achieved during the catch-up phase was maintained by circumcising cohorts as they entered the 15–49 year old population. The model assumed scale-up of VMMC at a fixed rate—that is, the likelihood of any male within the targeted population to be circumcised was uniform. These VMMC intervention scenarios assume 80% VMMC coverage among 15–49 year old males per the WHO/UNAIDS recommendation and as adopted by the Government of Zambia [11, 12, 36]. VMMC coverage of 80% has been also adopted as the baseline of choice across countries in SSA in the ongoing modeling efforts for assessing VMMC program efficiency gains [38, 59]. The number of VMMCs needed to avert one HIV infection (effectiveness) of the VMMC intervention program was evaluated over an intermediate period (2010–2025) and a long-term horizon (2010–2035). All analyses were produced assuming no antiretroviral therapy (ART) scale-up.

#### Implications of including HIV-positive males

Three different VMMC program scenarios were modeled. To allow for comparative analyses, VMMC scale-up was assumed identical in these three scenarios, and as representative of actual scale-up as possible, apart from the inclusion criteria of HIV-positive males.

In the first scenario, the VMMC program circumcised only HIV-negative males. In the second scenario, the VMMC program circumcised both HIV-negative and HIV-positive males, in line with program data for Zambia [37]. Accordingly, less than 1% of those circumcised in this scenario were HIV-positive. In the third scenario, the VMMC program circumcised males regardless of their HIV status (no differential targeting by HIV status), such that HIV-negative and -positive males had an equal chance of being circumcised. Accordingly, 12.6% of those circumcised in 2010 in this scenario were HIV-positive, similar to HIV prevalence among males of 12.6% in 2010. This fraction, however, changed in the simulations with the temporal evolution of HIV prevalence (S2 Fig). In the three scenarios, it was assumed that there was no effect on higher-risk males accepting services (does not dissuade higher-risk HIV-negative and -positive males from accepting VMMC).

#### Uptake among higher-risk males due to VMMC implementation criteria

The inclusivity of HIV-positive males in VMMC programs may affect the uptake of circumcision among higher-risk HIV-negative males, as they may avoid going to a VMMC program due to fear of being required to test for HIV, or learning their HIV status, or of testing positive for HIV [16, 17, 60, 61]. Increased VMMC uptake among higher-risk males may reduce the number of VMMCs needed to avert one HIV infection, since higher-risk males derive a greater absolute reduction in HIV acquisition and transmission risk than do lower-risk males [10, 38, 39]. For example, in our model application for Zambia, around 40% of HIV acquisitions and more than 90% of HIV transmissions are occurring among or through the three highest risk groups.

Against this background, we examined the impact of VMMC uptake among higher-risk HIV-negative males under different assumptions. Given that there are no specific quantitative data on the risk behavior distribution of males seeking circumcision, we conducted the following analyses:

- We modeled an intervention scenario in which we assessed the effectiveness of a VMMC program including only HIV-negative males, but with 20% lower male circumcision uptake among higher-risk males (the three highest risk groups in the model). The 20% lower uptake scenario among higher risk males was based on consensus of expert opinion.
- We modeled an intervention scenario in which we assessed the effectiveness of a VMMC program that includes HIV-positive males at a rate proportional to their representation in the population (e.g. 12.6% of those circumcised in 2010 would be HIV-positive males based on an HIV prevalence of 12.6% among males), but with 20% higher male circumcision uptake among higher-risk males. The 20% higher uptake scenario among higher risk males was based on consensus of expert opinion.

#### Efficacy of male circumcision against male-to-female HIV transmission

In our analyses, we used three different levels of efficacy of male circumcision against male-to-female HIV transmission:

1. No efficacy of male circumcision against male-to-female HIV transmission—i.e. 0%.
2. A 20% efficacy of male circumcision against male-to-female HIV transmission, as informed by the Weiss et al. systematic review and meta-analysis [19]
3. A 46% efficacy of male circumcision against male-to-female HIV transmission, as informed by the Hallett et al. meta-analysis of two quality measures [20]

We conducted a sensitivity analysis assessing the impact of higher HIV infectiousness among circumcised males who resume sexual activity before the complete healing of their circumcision wounds [18, 31, 62]. We assumed a 3.5-fold increase in male-to-female HIV transmission during the wound healing period—that is two to six weeks post circumcision—per available evidence [18, 34].

**Fig 1:**
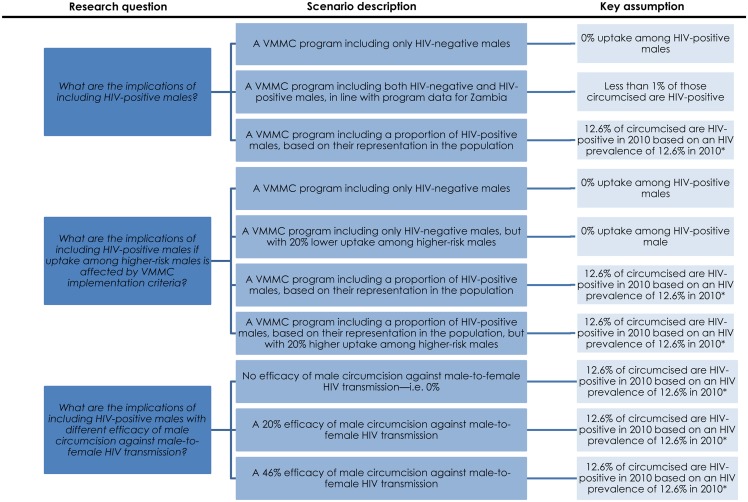
Diagram summarizing research questions and modeled intervention scenarios investigated in this study. * This fraction changes with the temporal changes in HIV prevalence Effectiveness is defined as the number of VMMCs needed to avert one HIV infection. VMMC: Voluntary medical male circumcision.

Fig 1 summarizes all of the above-modeled intervention scenarios.

### Uncertainty Analysis

Multivariate uncertainty analysis was conducted to specify the range of uncertainty in the effectiveness of VMMC with respect to variations in the structural and biological parameters of the model. The analysis was conducted using Monte Carlo sampling from uniform probability distributions (MATLAB algorithm: *unifrnd*) for the uncertainty in HIV natural history and transmission parameters and behavioral parameters of the model, and by assuming an uncertainty of 20% around the point estimates of all parameters. The varied parameters included: HIV transmission probability per coital act, duration of each HIV stage, frequency of coital acts per HIV stage, duration of partnership, degree of assortativeness, scale parameter in the gamma distribution of the population across the risk groups, and exponent parameter in the power-law function of the distribution of sexual-risk behavior (S2 Table). The uncertainty range of ±20% in input parameters was informed by the range used in modeling literature [45, 63, 64]. Each set of new parameters was used to refit the HIV prevalence time series data in Zambia and then assess the effectiveness of the VMMC program. We implemented 500 uncertainty runs for each modeled intervention scenario. The 500 runs were chosen as the optimal number of uncertainty runs encompassing as much as possible of the parameter space while maintaining computational feasibility. We derived the geometric mean effectiveness and associated 95% uncertainty interval from the distribution of outcomes of the uncertainty runs.

## Results

### Implications of Including HIV-Positive Males

**Fig 2:**
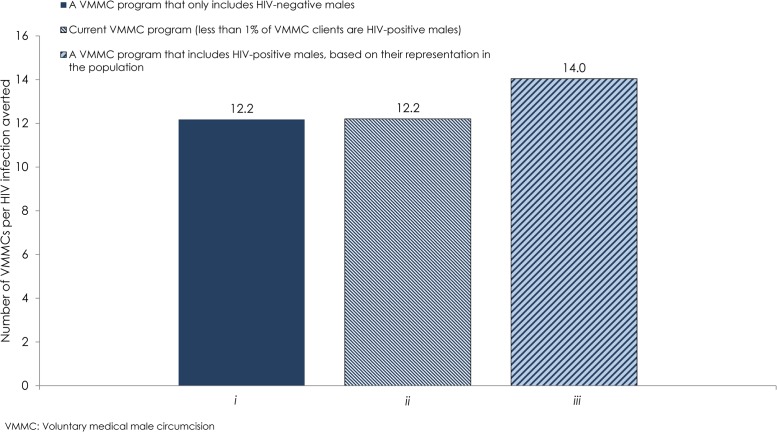
Impact of excluding or including HIV-positive males in the VMMC program in Zambia. The figure shows the effectiveness of the VMMC program including *i*) only HIV-negative males, *ii*) HIV-positive males as per current program data, and *iii*) proportion of HIV-positive males, based on their representation in the population. No efficacy of male circumcision against male-to-female HIV transmission was assumed. Effectiveness is defined as the number of VMMCs needed to avert one HIV infection.

Fig 2 shows three modeled VMMC intervention scenarios that assess the implications of including HIV-positive males in the VMMC program in Zambia. In these scenarios, no efficacy of male circumcision against male-to-female HIV transmission was assumed. In the first scenario, circumcising only HIV-negative males, the effectiveness was 12.2 VMMCs per HIV infection averted. In the second scenario, circumcising in accord with current program data (less than 1% of those circumcised are HIV-positive), the effectiveness was the same: 12.2 VMMCs per HIV infection averted. In the third scenario, circumcising males regardless of their HIV status, the effectiveness was 14.0 VMMCs per HIV infection averted.

S3 Fig shows the range of uncertainty for the three modeled VMMC intervention scenarios that assess the implications of including HIV-positive males in the VMMC program in Zambia. In each uncertainty run, the relative difference in VMMC effectiveness across scenarios was preserved confirming our results reported above.

### Uptake among Higher-Risk Males Due to VMMC Implementation Criteria

**Fig 3:**
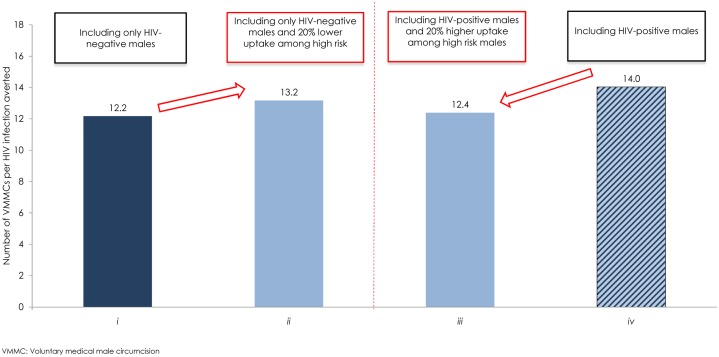
Effectiveness of the VMMC program in Zambia at different uptake levels among higher-risk males. The figure shows the effectiveness of the VMMC program including *i*) only HIV-negative males, *ii*) only HIV-negative males, but with 20% lower uptake of VMMC among higher-risk males, *iii*) proportion of HIV-positive males, based on their representation in the population, but with 20% higher uptake of VMMC among higher-risk males, and *iv*) proportion of HIV-positive males, based on their representation in the population. No efficacy of male circumcision against male-to-female HIV transmission was assumed. Effectiveness is defined as the number of VMMCs needed to avert one HIV infection.

Fig 3 shows the impact on Zambia’s VMMC program of excluding HIV-positive males, if the exclusion was associated with lower uptake of the VMMC intervention among higher-risk males. Here, no efficacy of male circumcision against male-to-female HIV transmission was assumed. Circumcising only HIV-negative males, the effectiveness was 12.2 VMMCs per HIV infection averted. In the scenario in which the VMMC program excluded HIV-positive males, but with 20% lower uptake among higher-risk males, the effectiveness was 13.2 VMMCs per HIV infection averted.

Fig 3 further shows the impact on Zambia’s VMMC program of including HIV-positive males, if the inclusion was associated with higher uptake of the VMMC intervention among higher-risk males. Circumcising regardless of HIV status, the effectiveness was 14.0 VMMCs per HIV infection averted. In the scenario in which the VMMC program included HIV-positive males, but with 20% higher uptake among higher-risk males, the effectiveness was 12.4 VMMCs per HIV infection averted.

### Efficacy of Male Circumcision against Male-to-Female HIV Transmission

**Fig 4:**
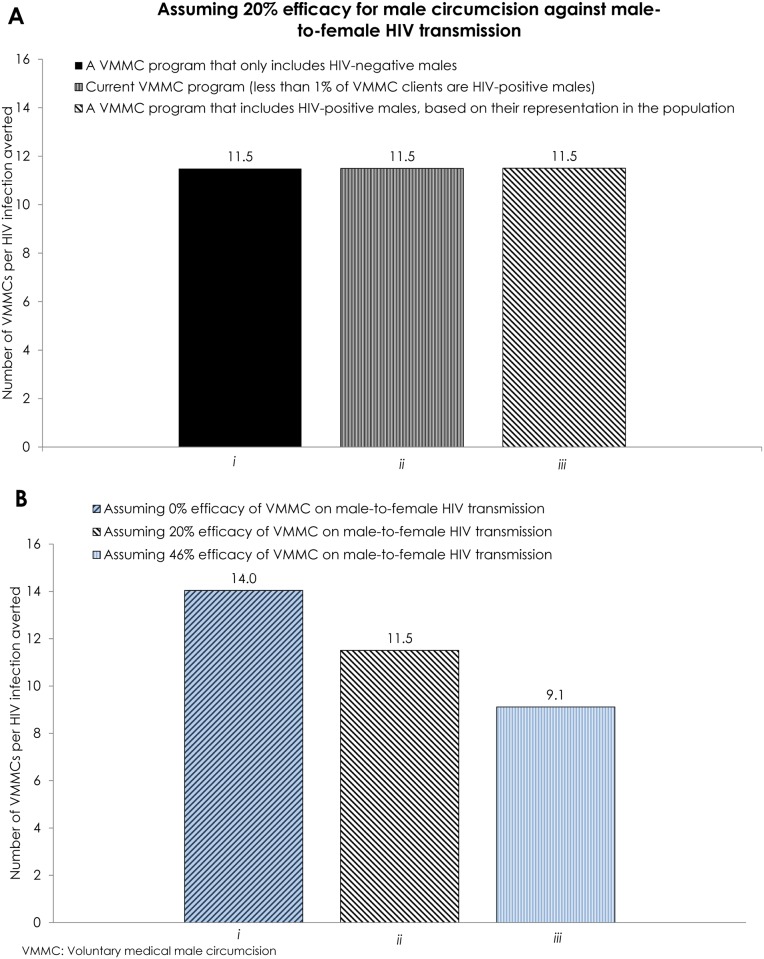
Effectiveness of the VMMC program in Zambia assuming different male circumcision efficacy levels against male-to-female HIV transmission. **A)** The panel shows the effectiveness of the VMMC program, assuming 20% efficacy for male circumcision against male-to-female HIV transmission, including *i*) only HIV-negative males, *ii*) HIV-positive males as per current program data, and *iii*) proportion of HIV-positive males, based on their representation in the population. **B)** The panel shows the effectiveness of the VMMC program including a proportion of HIV-positive males, based on their representation in the population, but assuming *i*) 0% efficacy, *ii*) 20% efficacy, and *iii*) 46% efficacy against male-to-female HIV transmission. Effectiveness is defined as the number of VMMCs needed to avert one HIV infection.

Fig 4 shows the impact of assuming an efficacy for male circumcision against male-to-female HIV transmission. Assuming 20% efficacy, the effectiveness was 11.5 VMMCs per HIV infection averted, regardless of scenario: circumcising only HIV-negative males, circumcising males in accord with current program data, or circumcising males regardless of their HIV status (Fig 4A). By incorporating an efficacy of VMMC against HIV acquisition among circumcised HIV negative males and a 20% efficacy of VMMC against male-to-female HIV transmission among circumcised HIV positive males, the model produced (after rounding) similar quantitative results in the three scenarios regardless of inclusion criteria of HIV-positive males.

Fig 4B shows the effectiveness of the VMMC program at different male circumcision efficacy levels of 0%, 20%, and 46%. In these scenarios, males were circumcised regardless of their HIV status. At 0% efficacy, the effectiveness was 14.0 VMMCs per HIV infection averted. At 20% efficacy, the effectiveness was 11.5 VMMCs per HIV infection averted. At 46% efficacy, the effectiveness was 9.1 VMMCs per HIV infection averted.

S4 Fig shows the sensitivity analysis assessing the impact of higher HIV infectiousness among circumcised males who resume sexual activity before the complete healing of their circumcision wound. The higher HIV infectiousness marginally affected both VMMC effectiveness (comparing S4 Fig to Fig 2) and the relative differences in VMMC effectiveness (comparing the three scenarios in S4 Fig to each other).

### The Impact of Male Circumcision Efficacy Levels on the HIV Incidence Rate

**Fig 5:**
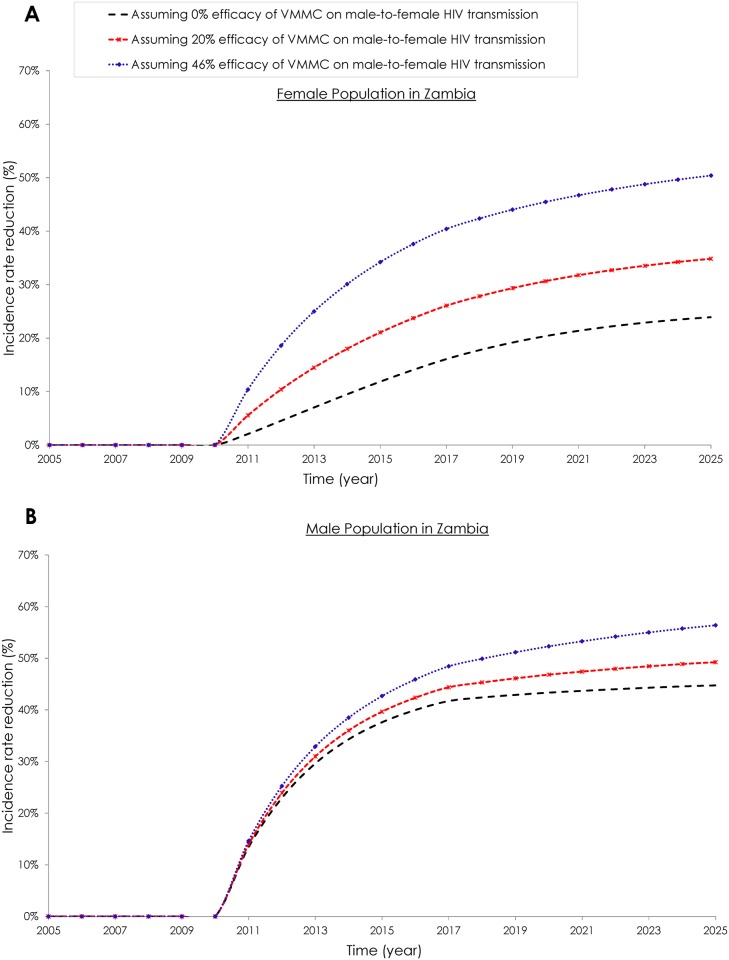
Impact of different male circumcision efficacy levels, against male-to-female HIV transmission, on HIV incidence rate reduction in Zambia. Incidence rate reduction in **A)** the female population and in **B)** the male population with the VMMC program including a proportion of HIV-positive males, based on their representation in the population. The panels assume *i*) 0% efficacy, *ii*) 20% efficacy, and *iii*) 46% efficacy of male circumcision against male-to-female HIV transmission.

Fig 5 shows the impact on the HIV incidence rate of different male circumcision efficacy levels against male-to-female HIV transmission. In these scenarios, males were circumcised regardless of their HIV status, assuming no effect on VMMC uptake among higher-risk HIV-negative males. At 0% efficacy, the incidence rate reduction among the female population in Zambia was 24.7% in the year 2025 (Fig 5A). At 20% efficacy, the incidence rate reduction was 34.8% that same year, and at 46% efficacy, the incidence rate reduction was 50.4% (Fig 5A).

At 0% efficacy, the incidence rate reduction among the male population in Zambia was 45.2% in the year 2025 (Fig 5B). At 20% efficacy, the incidence rate reduction was 49.2% that same year, and at 46% efficacy, the incidence rate reduction was 56.4% (Fig 5B).

Table 1 summarizes the results of all of the above-modeled intervention scenarios evaluated over an intermediate horizon (2010–2025), while S2 Table summarizes the results of all of the above-modeled intervention scenarios evaluated over a long-term horizon (2010–2035).

**Table 1:** Effectiveness of the VMMC program in Zambia under different VMMC intervention scenarios.

Modeled VMMC intervention scenario	Assumed efficacy of male circumcision against male-to-female HIV transmission	Effectiveness (number of VMMCs per HIV infection averted)
*Implications of including HIV-positive males* (Fig 2)		
A VMMC program including only HIV-negative males	0%	12.2
Current VMMC program (less than 1% of those circumcised are HIV-positive)	0%	12.2
A VMMC program including a proportion of HIV-positive males, based on their representation in the population*	0%	14.0
*Uptake among higher-risk males due to VMMC implementation criteria* (Fig 3)		
A VMMC program including only HIV-negative males	0%	12.2
A VMMC program including only HIV-negative males, but with 20% lower uptake among higher-risk males	0%	13.2
A VMMC program including a proportion of HIV-positive males, based on their representation in the population*	0%	14.0
A VMMC program including a proportion of HIV-positive males, based on their representation in the population*, but with 20% higher uptake among higher-risk males	0%	12.4
*Efficacy of male circumcision against male-to-female HIV transmission* (Fig 4)		
Fig 4A		
A VMMC program including only HIV-negative males	20%	11.5
Current VMMC program (less than 1% of those circumcised are HIV-positive)	20%	11.5
A VMMC program including a proportion of HIV-positive males, based on their representation in the population*	20%	11.5
Fig 4B		
A VMMC program including a proportion of HIV-positive males, based on their representation in the population*	0%	14.0
A VMMC program including a proportion of HIV-positive males, based on their representation in the population*	20%	11.5
A VMMC program including a proportion of HIV-positive males, based on their representation in the population*	46%	9.1

Effectiveness is defined as the number of VMMCs needed to avert one HIV infection. VMMC: Voluntary medical male circumcision.

* For instance, 12.6% of those circumcised in 2010 were HIV-positive based on an HIV prevalence of 12.6% among males in 2010. This percentage changes with the temporal changes in HIV prevalence in Zambia.

## Discussion

Epidemiological and some programmatic implications of including HIV-positive males in VMMC programs were examined in this study using mathematical modeling (the ASM model). The primary finding is that including HIV-positive males in VMMC programs is unlikely to substantially undermine the efficiency of VMMC programs to achieve the desired epidemic impact. On the contrary, implementing VMMCs regardless of HIV status may improve the effectiveness of VMMC programs, by reducing the number of VMMCs needed to avert one HIV infection, depending on the assumptions.

Of all of the scenarios considered, only the one that assumed 0% protection against male-to-female transmission and no effect on uptake among higher-risk males resulted in lower effectiveness by including HIV-positive males. Although a VMMC program in Zambia that reaches only HIV-negative males may reduce the number of VMMCs per HIV infection averted by up to 15% (Fig 2), in reality, this is not likely to occur. Though not yet validated with concrete data, we believe that programs that inadvertently reach only HIV-negative males may be discouraging higher-risk males from presenting for VMMC due to concerns about mandatory HTC and knowledge or disclosure of their HIV status. Decreased demand for services among these males could lower the effectiveness of the VMMC programs. If VMMC uptake among higher-risk HIV-negative males is reduced by as little as 20%, the number of VMMCs needed to avert one HIV infection would increase by 8% (Fig 3). If VMMC uptake among higher-risk HIV-negative males is reduced by more than 20%, the number of VMMCs needed to avert one HIV infection would increase even more.

If, on the other hand, improved inclusivity of HIV-positive males increases VMMC uptake among higher-risk males, the increased effectiveness will likely make inclusion of HIV-positive males more beneficial than exclusion. For example, if VMMC uptake among higher-risk males is increased by as little as 20% (in a program that includes HIV-positive males), the number of VMMCs needed to avert one HIV infection will be similar to that of a program that only circumcises HIV-negative males (Fig 3). If VMMC uptake among higher-risk males is increased by more than 20%, the number of VMMCs needed to avert one HIV infection will decrease even more.

These results are not surprising in light of recent findings showing that the number of VMMCs needed to avert one HIV infection is highly sensitive to risk-group targeting [38]. Higher uptake among higher-risk males leads to a greater absolute reduction in risk and greater epidemiological impact in terms of averted HIV infections. This finding, we believe, hints indirectly to the importance of reducing HIV stigma and avoiding lower uptake of VMMC among high-risk males. We believe that recruitment messages should frame VMMC more broadly as a sexual and reproductive health service, reinforce that HTC services are offered but not required, and ensure health care workers are providing VMMC to clinically eligible males regardless of HIV status and willingness to test for HIV.

Furthermore, if male circumcision has any efficacy against male-to-female HIV transmission—an effect for which there is empirical evidence [19, 20, 65]—the number of VMMCs needed to avert one HIV infection will be further reduced by inclusion of HIV-positive males. If the efficacy of circumcision against male-to-female HIV transmission is as little as 20%, the number of VMMCs needed per HIV infection averted, in a VMMC program that circumcises HIV-positive males at a rate proportional to their representation in the population, would be reduced by 22% (Fig 4A). If the efficacy is as high as 46%, the number of VMMCs needed per HIV infection averted would be reduced by 55% (Fig 4B).

The combination of a moderate increase in VMMC uptake among higher-risk males (i.e. 20%), and a moderate efficacy of male circumcision against male-to-female HIV transmission (i.e. 20%), supports program efficiency of including HIV-positive males in VMMC programs. Inclusion of HIV-positive males in VMMC programs would reduce HIV incidence rate by as much as some lifelong ART programs achieve. ART coverage in some programs can reduce HIV incidence rate by as much as 50% [66], while our results show that VMMC (a one-time intervention) can reduce HIV incidence rate by as much as 42%.

The evidence of male circumcision efficacy against male-to-female HIV transmission has an important implication: VMMC programs will benefit females nearly as much as males. If VMMC’s efficacy against male-to-female HIV transmission is 46%, as a recent meta-analysis found [20], the incidence rate reduction in 2025 would be 50% among females, in comparison to 56% among males (Fig 5). Uncircumcised males will also benefit if such efficacy exists, though indirectly and at lower levels. The reduction in HIV incidence rate in 2025 among uncircumcised males in the population, regardless of circumcision status, would be 16% at 0% efficacy in comparison with 36% at 46% efficacy (data not shown).

## Limitations

Our results may depend on the type of mathematical model structure used and input parameters incorporated in the model. The availability of precise and representative time-trend data for HIV prevalence, baseline male circumcision prevalence, and demographic data such as total population size and future projections is critical for the formulation and examination of the modeled intervention scenarios. In the modeled intervention scenarios, we assumed a fixed VMMC rate in all scenarios, which does not precisely capture the current VMMC scale-up rate for Zambia. We assumed that exclusion or inclusion of HIV-positive males in VMMC programs may change uptake among higher-risk males by as much as 20%. However, there are no empirical data reporting how exactly uptake changes with exclusion or inclusion of HIV-positive males. In the intervention scenarios tested here, we assumed different levels of efficacy for male circumcision against male-to-female HIV transmission. Nonetheless, a conclusive estimate of the size of this protective effect is still lacking.

Despite these potential limitations, we used an elaborate mathematical model structure (the ASM model) that can capture the complexity of different effects of VMMC programs on HIV dynamics (S1 Text) [38]. Among these complexities is the effect of VMMC intervention on onward HIV transmission—that is the indirect effect of VMMC on females by reducing HIV incidence among circumcised males. This model is an extension of earlier models that were developed and refined over the years to study HIV epidemic dynamics in SSA [45, 67, 68]. We conducted a multivariate uncertainty analysis on our model predictions to assess the impact of changes in the structural, behavioral and biological parameters of the model. This analysis affirmed the validity of our predictions (S3 Fig). Our model was parameterized by current empirical epidemiological data on HIV infection in SSA, and particularly in Zambia, though most of the biological data were from other SSA countries and they may not represent data from Zambia if such data will become available. Although we used a fixed scale-up rate rather than Zambia’s precise (variable) VMMC scale-up rate, our earlier modeling study showed that the impact of the VMMC program using a variable VMMC scale-up rate was virtually similar to that with a fixed rate [38].

Scale-up of other interventions such as ART or condom distribution may potentially diminish the benefits of VMMC scale-up programs. However, our earlier modeling study showed that even with an optimistic ART scale-up scenario, VMMC in an HIV hyper-endemic setting such as Zambia will remain an impactful and effective intervention. Also, scale-up of ART is not likely to change our predictions of the programmatic implications of including HIV-positive males in VMMC programs, as our study focused on the relative differences in VMMC effectiveness across scenarios rather than the absolute changes in VMMC effectiveness.

In our model we did not explore other relevant components of VMMC efficiency such as that of reduction in HIV testing costs that may shift the trade-offs in balance of a broad recruitment strategy. Also, the implications of inclusion of HIV-positive males in VMMC programs was only assessed at a national level rather than at subregional and/or rural versus urban levels. Nonetheless, an earlier modeling study indicated that geographical heterogeneities do not have a considerable effect on the *relative* difference in VMMC effectiveness [38].

Furthermore, though we do not yet know the exact magnitude of the efficacy of male circumcision against male-to-female HIV transmission, a mounting body of evidence suggests that the effect is sizable [19, 20, 65]. To accommodate a range of predictions in our study, we assumed three different male circumcision efficacy levels. The results indicated that including HIV-positive males at a rate proportional to their representation in the population (e.g. 12.6% of those circumcised in 2010) is unlikely to undermine the effectiveness of VMMC programs (Fig 4). We also conducted a sensitivity analysis that accommodates for higher HIV infectiousness among circumcised males who resume their sexual activity before the complete healing of the circumcision wound (S4 Fig). The analysis indicated that resumption of sexual activity during the healing period of the circumcision wound will only minimally reduce the effectiveness of VMMC programs (compare results in S4 Fig with Fig 2), and only in the short term, as has been shown in earlier modeling studies [7, 8, 20, 25, 34].

## Conclusion

Improving the inclusivity of all males in VMMC programs is likely to enhance the programs’ effectiveness. Enhanced effectiveness would occur with moderate increases in the uptake of VMMC among males at higher risk and/or if male circumcision has moderate efficacy against male-to-female HIV transmission. In these circumstances, the one-time VMMC intervention can reduce the HIV incidence rate within a population by as much as some lifelong ART programs do. The reduction in incidence rate among females under these conditions can also be nearly as high as that among males.

In light of these results, VMMC programs may wish to attract males regardless of their willingness to test for HIV and learn their HIV status. Program planners should not hesitate to recruit VMMC clients among populations at higher risk for HIV infection for fear of inadvertently increasing participation by HIV-positive males. Indeed, we believe that restricting recruitment in this way could affect the uptake of VMMC among both HIV-positive and -negative males, which would undermine a program’s overall impact. We believe that recruiting clients regardless of their perceived HIV status or willingness to be tested could reduce stigma and increase uptake of VMMC among higher-risk males, leading to more effective VMMC programs. Better understanding of the factors that affect the uptake of VMMC among males will allow programs to overcome the plausible lower uptake among higher-risk males while maintaining their links to other HIV care and treatment services, such as HTC.

## Supporting Information

S1 Text Further Details on the Age-Structured Mathematical (ASM) Model. (DOCX)

S1 Table Definitions of the symbols in the equations of the age-structured mathematical (ASM) model. (DOCX)

S2 Table Model assumptions in terms of parameter values. (DOCX)

S3 Table Effectiveness of the VMMC program in Zambia by 2035 under different VMMC intervention scenarios. (DOCX)

S1 Fig Schematic illustration of HIV transmission dynamics with voluntary medical male circumcision as an HIV prevention intervention. (DOCX)

S2 Fig Model prediction of HIV prevalence in the 15–49 year old population in Zambia. (DOCX)

S3 Fig Range of uncertainty for the impact of excluding or including HIV-positive males in the VMMC program in Zambia. (DOCX)

S4 Fig Sensitivity analysis for the impact of increased risk for male-to-female HIV transmission among circumcised males who resume their sexual activity during the healing period. (DOCX)

## Abbreviations

ART: antiretroviral therapy
ASM: Age-Structured Mathematical
HTC: HIV testing and counseling
RCT: Randomized controlled trial
SSA: sub-Saharan Africa
UNAIDS: Joint United Nations Programme on HIV/AIDS
VMMC: voluntary medical male circumcision
WHO: World Health Organization

## References

[1] Auvert B, Taljaard D, Lagarde E, Sobngwi-Tambekou J, Sitta R, Puren A. Randomized, controlled intervention trial of male circumcision for reduction of HIV infection risk: the ANRS 1265 Trial. PLoS Med. 2005;2(11):e298 10.1371/journal.pmed.0020298 16231970 PMC1262556

[2] Bailey RC, Moses S, Parker CB, Agot K, Maclean I, Krieger JN, et al Male circumcision for HIV prevention in young men in Kisumu, Kenya: a randomised controlled trial. Lancet. 2007;369(9562):643–56. Epub 2007/02/27. 10.1016/S0140-6736(07)60312-2 17321310

[3] Gray RH, Kigozi G, Serwadda D, Makumbi F, Watya S, Nalugoda F, et al Male circumcision for HIV prevention in men in Rakai, Uganda: a randomised trial. Lancet. 2007;369(9562):657–66. Epub 2007/02/27. 10.1016/S0140-6736(07)60313-4 17321311

[4] Nagelkerke NJ, Moses S, de Vlas SJ, Bailey RC. Modelling the public health impact of male circumcision for HIV prevention in high prevalence areas in Africa. BMC Infect Dis. 2007;7:16 10.1186/1471-2334-7-16 17355625 PMC1832203

[5] Gray RH, Li X, Kigozi G, Serwadda D, Nalugoda F, Watya S, et al The impact of male circumcision on HIV incidence and cost per infection prevented: a stochastic simulation model from Rakai, Uganda. AIDS. 2007;21(7):845–50. 10.1097/QAD.0b013e3280187544 17415039

[6] Williams BG, Lloyd-Smith JO, Gouws E, Hankins C, Getz WM, Hargrove J, et al The potential impact of male circumcision on HIV in Sub-Saharan Africa. PLoS Med. 2006;3(7):e262 10.1371/journal.pmed.0030262 16822094 PMC1489185

[7] Hallett TB, Singh K, Smith JA, White RG, Abu-Raddad LJ, Garnett GP. Understanding the impact of male circumcision interventions on the spread of HIV in southern Africa. PLoS One. 2008;3(5):e2212 Epub 2008/05/22. 10.1371/journal.pone.0002212 18493593 PMC2387228

[8] Alsallaq RA, Cash B, Weiss HA, Longini IM Jr., Omer SB, Wawer MJ, et al Quantitative assessment of the role of male circumcision in HIV epidemiology at the population level. Epidemics. 2009;1(3):139–52. Epub 2009/09/01. 10.1016/j.epidem.2009.08.001 21352761

[9] White RG, Glynn JR, Orroth KK, Freeman EE, Bakker R, Weiss HA, et al Male circumcision for HIV prevention in sub-Saharan Africa: who, what and when? AIDS. 2008;22(14):1841–50. Epub 2008/08/30. 10.1097/QAD.0b013e32830e0137 18753931

[10] Njeuhmeli E, Forsythe S, Reed J, Opuni M, Bollinger L, Heard N, et al Voluntary medical male circumcision: modeling the impact and cost of expanding male circumcision for HIV prevention in eastern and southern Africa. PLoS Med. 2011;8(11):e1001132 Epub 2011/12/06. 10.1371/journal.pmed.1001132 22140367 PMC3226464

[11] WHO and Joint United Nations Programme on HIV/AIDS (UNAIDS). New data on male circumcision and HIV prevention: policy and programme implications. WHO/UNAIDS technical consultation on male circumcision and HIV prevention: research implications for policy and programming. Geneva, Switzerland: WHO. March 6–8, 2007.

[12] World Health Organization (WHO). Joint strategic action framework to accelerate the scale-up of voluntary medical male circumcision for HIV prevention in Eastern and Southern Africa. Geneva: 2011.

[13] Marks G, Crepaz N, Janssen RS. Estimating sexual transmission of HIV from persons aware and unaware that they are infected with the virus in the USA. AIDS. 2006;20(10):1447–50 10.1097/01.aids.0000233579.79714.8d 16791020

[14] Dokubo EK, Shiraishi RW, Young PW, Neal JJ, Aberle-Grasse J, Honwana N, et al Awareness of HIV status, prevention knowledge and condom use among people living with HIV in Mozambique. PLoS One. 2014;9(9):e106760 10.1371/journal.pone.0106760 25222010 PMC4164358

[15] Kikaya V, Skolnik L, Garcia MC, Nkonyana J, Curran K, Ashengo TA. Voluntary medical male circumcision programs can address low HIV testing and counseling usage and ART enrollment among young men: lessons from Lesotho. PLoS One. 2014;9(5):e83614 10.1371/journal.pone.0083614 24801714 PMC4011866

[16] George G, Strauss M, Chirawu P, Rhodes B, Frohlich J, Montague C, et al Barriers and facilitators to the uptake of voluntary medical male circumcision (VMMC) among adolescent boys in KwaZulu-Natal, South Africa. Afr J AIDS Res. 2014;13(2):179–87. 10.2989/16085906.2014.943253 25174635

[17] Hatzold K, Mavhu W, Jasi P, Chatora K, Cowan FM, Taruberekera N, et al Barriers and motivators to voluntary medical male circumcision uptake among different age groups of men in Zimbabwe: results from a mixed methods study. PLoS One. 2014;9(5):e85051 10.1371/journal.pone.0085051 24802746 PMC4011705

[18] Wawer MJ, Makumbi F, Kigozi G, Serwadda D, Watya S, Nalugoda F, et al Circumcision in HIV-infected men and its effect on HIV transmission to female partners in Rakai, Uganda: a randomised controlled trial. Lancet. 2009;374(9685):229–37. Epub 2009/07/21. 10.1016/S0140-6736(09)60998-3 19616720 PMC2905212

[19] Weiss HA, Hankins CA, Dickson K. Male circumcision and risk of HIV infection in women: a systematic review and meta-analysis. Lancet Infect Dis. 2009;9(11):669–77. Epub 2009/10/24. 10.1016/S1473-3099(09)70235-X 19850225

[20] Hallett TB, Alsallaq RA, Baeten JM, Weiss H, Celum C, Gray R, et al Will circumcision provide even more protection from HIV to women and men? New estimates of the population impact of circumcision interventions. Sex Transm Infect. 2011;87(2):88–93. Epub 2010/10/23. 10.1136/sti.2010.043372 20966458 PMC3272710

[21] Baeten JM, Donnell D, Kapiga SH, Ronald A, John-Stewart G, Inambao M, et al Male circumcision and risk of male-to-female HIV-1 transmission: a multinational prospective study in African HIV-1-serodiscordant couples. AIDS. 2010;24(5):737–44. Epub 2010/01/01. 10.1097/QAD.0b013e32833616e0 20042848 PMC2919808

[22] Turner AN, Morrison CS, Padian NS, Kaufman JS, Salata RA, Chipato T, et al Men's circumcision status and women's risk of HIV acquisition in Zimbabwe and Uganda. AIDS. 2007;21(13):1779–89. Epub 2007/08/11. 10.1097/QAD.0b013e32827b144c 17690577 PMC2978032

[23] Gray RH, Kiwanuka N, Quinn TC, Sewankambo NK, Serwadda D, Mangen FW, et al Male circumcision and HIV acquisition and transmission: cohort studies in Rakai, Uganda. Rakai Project Team. AIDS. 2000;14(15):2371–81. Epub 2000/11/23. 11089626 10.1097/00002030-200010200-00019

[24] Kapiga SH, Lyamuya EF, Lwihula GK, Hunter DJ. The incidence of HIV infection among women using family planning methods in Dar es Salaam, Tanzania. AIDS. 1998;12(1):75–84. Epub 1998/02/10. 9456257 10.1097/00002030-199801000-00009

[25] Kamath V, Limaye RJ. Voluntary medical male circumcision for HIV prevention and early resumption of sexual activity: a literature review. AIDS Care. 2015:1–4. 10.1080/09540121.2015.1017797 25738780

[26] Allen S, Lindan C, Serufilira A, Van de Perre P, Rundle AC, Nsengumuremyi F, et al Human immunodeficiency virus infection in urban Rwanda. Demographic and behavioral correlates in a representative sample of childbearing women. JAMA. 1991;266(12):1657–63. Epub 1991/09/25. 1886188

[27] Quinn TC, Wawer MJ, Sewankambo N, Serwadda D, Li CJ, Wabwire-Mangen F, et al Viral load and heterosexual transmission of human immunodeficiency virus type 1. New England Journal of Medicine. 2000;342(13):921–9. 10.1056/NEJM200003303421303 10738050

[28] Gray R, Thoma M, Laeyendecker O, Serwadda D, Nalugoda F, Kigozi G, et al. Male Circumcision and the Risks of Female HIV and STI Acquisition in Rakai, Uganda. Presentation at the 2006 Conference on Retroviruses and Opportunistic Infections (CROI), Feb 8, Denver Colorado. Press. Available: www.hopkinsmedicine.org/Press_releases/2006/02_08_06.html. 2006.

[29] Malamba SS, Mermin JH, Bunnell R, Mubangizi J, Kalule J, Marum E, et al Couples at risk: HIV-1 concordance and discordance among sexual partners receiving voluntary counseling and testing in Uganda. J Acquir Immune Defic Syndr. 2005;39(5):576–80. Epub 2005/07/27. 16044010

[30] Chao A, Bulterys M, Musanganire F, Habimana P, Nawrocki P, Taylor E, et al Risk factors associated with prevalent HIV-1 infection among pregnant women in Rwanda. National University of Rwanda-Johns Hopkins University AIDS Research Team. Int J Epidemiol. 1994;23(2):371–80. Epub 1994/04/01. 8082965 10.1093/ije/23.2.371

[31] Mehta SD, Gray RH, Auvert B, Moses S, Kigozi G, Taljaard D, et al Does sex in the early period after circumcision increase HIV-seroconversion risk? Pooled analysis of adult male circumcision clinical trials. AIDS. 2009;23(12):1557–64. Epub 2009/07/03. 10.1097/QAD.0b013e32832afe95 19571722 PMC2772053

[32] Kigozi G, Gray RH, Wawer MJ, Serwadda D, Makumbi F, Watya S, et al The safety of adult male circumcision in HIV-infected and uninfected men in Rakai, Uganda. PLoS Med. 2008;5(6):e116 10.1371/journal.pmed.0050116 18532873 PMC2408615

[33] Herman-Roloff A, Bailey RC, Agot K. Factors associated with the early resumption of sexual activity following medical male circumcision in Nyanza province, Kenya. AIDS Behav. 2012;16(5):1173–81. 10.1007/s10461-011-0073-1 22052231 PMC3677080

[34] Hewett PC, Hallett TB, Mensch BS, Dzekedzeke K, Zimba-Tembo S, Garnett GP, et al Sex with stitches: assessing the resumption of sexual activity during the postcircumcision wound-healing period. AIDS. 2012;26(6):749–56. 10.1097/QAD.0b013e32835097ff 22269970

[35] UNAIDS. Ending the AIDS epidemic by 2030. Reducing sexual transmission Geneva:UNAIDS: 2014. http://www.unaids.org/sites/default/files/media_asset/20140411_MeetingReport_Reducingsexualtransmission_en.pdf.

[36] Republic of Zambia Ministry of Health. Country Operational Plan for the Scale-up of Voluntary Medical Male Circumcision in Zambia, 2012–2015, http://www.malecircumcision.org/country_updates/documents/Zambia_VMMC_operational_plan.pdf. 2012.

[37] Sgaier SK. Performed voluntary medical male circumcision data for Zambia (2007–2014). Country-level data, Lusaka, Zambia. 2014.

[38] Awad SF, Sgaier SK, Tambatamba BC, Mohamoud YA, Lau FK, Reed JB, et al Investigating Voluntary Medical Male Circumcision Program Efficiency Gains through Subpopulation Prioritization: Insights from Application to Zambia. PLoS One. 2015;10(12):e0145729 10.1371/journal.pone.0145729 26716442 PMC4696770

[39] Awad SF, Sgaier SK, Ncube G, Xaba S, Mugurungi OM, Mhangara MM, et al A Reevaluation of the Voluntary Medical Male Circumcision Scale-Up Plan in Zimbabwe. PLoS One. 2015;10(10):e0140818. 26529596 10.1371/journal.pone.0140818 PMC4646702

[40] Cuadros DF, Crowley PH, Augustine B, Stewart SL, Garcia-Ramos G. Effect of variable transmission rate on the dynamics of HIV in sub-Saharan Africa. BMC Infect Dis. 2011;11:216 Epub 2011/08/13. 10.1186/1471-2334-11-216 21834977 PMC3175213

[41] Handcock MS, Jones JH. Likelihood-based inference for stochastic models of sexual network formation. Theor Popul Biol. 2004;65(4):413–22. Epub 2004/05/12. 10.1016/j.tpb.2003.09.006 15136015

[42] Hamilton DT, Handcock MS, Morris M. Degree distributions in sexual networks: a framework for evaluating evidence. Sex Transm Dis. 2008;35(1):30–40. Epub 2008/01/25. 18217224 10.1097/olq.0b013e3181453a84 PMC4370286

[43] Bansal S, Grenfell BT, Meyers LA. When individual behaviour matters: homogeneous and network models in epidemiology. J R Soc Interface. 2007;4(16):879–91. Epub 2007/07/21. 10.1098/rsif.2007.1100 17640863 PMC2394553

[44] Omori R, Chemaitelly H, Abu-Raddad LJ. Dynamics of non-cohabiting sex partnering in sub-Saharan Africa: a modelling study with implications for HIV transmission. Sex Transm Infect. 2015;91(6):451–7. 10.1136/sextrans-2014-051925 25746040 PMC4552955

[45] Awad SF, Abu-Raddad LJ. Could there have been substantial declines in sexual risk behavior across sub-Saharan Africa in the mid-1990s? Epidemics. 2014;8:9–17. 10.1016/j.epidem.2014.06.001 25240899

[46] Gregson S, Nyamukapa CA, Garnett GP, Mason PR, Zhuwau T, Carael M, et al Sexual mixing patterns and sex-differentials in teenage exposure to HIV infection in rural Zimbabwe. Lancet. 2002;359(9321):1896–903. Epub 2002/06/12. 10.1016/S0140-6736(02)08780-9 12057552

[47] Chemaitelly H, Cremin I, Shelton J, Hallett TB, Abu-Raddad LJ. Distinct HIV discordancy patterns by epidemic size in stable sexual partnerships in sub-Saharan Africa. Sex Transm Infect. 2012;88(1):51–7. Epub 2012/01/18. 10.1136/sextrans-2011-050114 22250180 PMC3261749

[48] Central Statistical Office MoH, University of Zambia, Lusaka, Zambia, Tropical Diseases Research Centre, Ndola, Zambia and Macro International Inc. Calverton, Maryland, USA Zambia DHS, 2007—Final Report; http://www.measuredhs.com/pubs/pdf/FR211/FR211[revised-05-12-2009].pdf. 2009.

[49] Westercamp N, Agot K, Jaoko W, Bailey RC. Risk compensation following male circumcision: results from a two-year prospective cohort study of recently circumcised and uncircumcised men in Nyanza Province, Kenya. AIDS Behav. 2014;18(9):1764–75. Epub 2014/07/23. 10.1007/s10461-014-0846-4 25047688

[50] Rosario IJ, Kasabwala K, Sadeghi-Nejad H. Circumcision as a strategy to minimize HIV transmission. Current urology reports. 2013;14(4):285–90. Epub 2013/06/19. 10.1007/s11934-013-0343-8 23775468

[51] UNAIDS. Political declaration on HIV and AIDS: Intensifying our efforts to eliminate HIV and AIDS. Geneva: UNAIDS: 2011.

[52] UNAIDS. Global AIDS response progress reporting 2012: guidelines—construction of core indicators for monitoring the 2011 political declaration on HIV/AIDS. Geneva: UNAIDS: 2012.

[53] UNAIDS. UNAIDS report on the global AIDS epidemic-2012. Annual UNAIDS report on the status of the AIDS epidemic and update on global initiatives to control it. Geneva: UNAIDS: 2012.

[54] UNAIDS. Epidemiological data, HIV estimates 1990–2013. 2013. http://www.unaids.org/en/dataanalysis/datatools/aidsinfo.

[55] Zambia Demographic and Health Survey 2007. http://dhsprogram.com/pubs/pdf/FR211/FR211%5Brevised-05-12-2009%5D.pdf [Internet]. CSO and Macro International Inc. 2009.

[56] United Nations Department of Economic and Social Affairs, Population Division, Population Estimates and Projections Section. World population prospects, the 2012 revision. 2012. http://esa.un.org/wpp/Excel-Data/population.htm

[57] The MathWorks, Inc. MATLAB. The language of technical computing. 8.5.0.197613 (R2015a). Natick, MA, USA: ed: The MathWorks, Inc.; 2015.

[58] Lagarias JC, Reeds J. A., Wright M. H. and Wright P. E.. Convergence Properties of the Nelder-MeadSimplex Method in Low Dimensions. SIAM Journal of Optimization. 1998;9(1):112–47.

[59] Sgaier SK, Reed JB, Thomas A, Njeuhmeli E. Achieving the HIV prevention impact of voluntary medical male circumcision: lessons and challenges for managing programs. PLoS Med. 2014;11(5):e1001641 Epub 2014/05/08. 10.1371/journal.pmed.1001641 24800840 PMC4011573

[60] Weiser SD, Heisler M, Leiter K, Percy-de Korte F, Tlou S, DeMonner S, et al Routine HIV testing in Botswana: a population-based study on attitudes, practices, and human rights concerns. PLoS Med. 2006;3(7):e261 10.1371/journal.pmed.0030261 16834458 PMC1502152

[61] Layer EH, Kennedy CE, Beckham SW, Mbwambo JK, Likindikoki S, Davis WW, et al Multi-level factors affecting entry into and engagement in the HIV continuum of care in Iringa, Tanzania. PLoS One. 2014;9(8):e104961 10.1371/journal.pone.0104961 25119665 PMC4138017

[62] Tobian AA, Kigozi G, Manucci J, Grabowski MK, Serwadda D, Musoke R, et al HIV shedding from male circumcision wounds in HIV-infected men: a prospective cohort study. PLoS Med. 2015;12(4):e1001820 10.1371/journal.pmed.1001820 25919012 PMC4412625

[63] Chemaitelly H, Awad SF, Shelton JD, Abu-Raddad LJ. Sources of HIV incidence among stable couples in sub-Saharan Africa. Journal of the International AIDS Society. 2014;17:18765 Epub 2014/02/25. 10.7448/IAS.17.1.18765 24560339 PMC3935448

[64] Chemaitelly H, Awad SF, Abu-Raddad LJ. The risk of HIV transmission within HIV-1 sero-discordant couples appears to vary across sub-Saharan Africa. Epidemics. 2014;6:1–9. Epub 2014/03/07. 10.1016/j.epidem.2013.11.001 24593916

[65] Auvert B. Efficacy of male circumcision against male-to-female transmission. Personal communication. 2014.

[66] Eaton JW, Johnson LF, Salomon JA, Barnighausen T, Bendavid E, Bershteyn A, et al HIV treatment as prevention: systematic comparison of mathematical models of the potential impact of antiretroviral therapy on HIV incidence in South Africa. PLoS Med. 2012;9(7):e1001245 10.1371/journal.pmed.1001245 22802730 PMC3393664

[67] Abu-Raddad LJ, Longini IM Jr. No HIV stage is dominant in driving the HIV epidemic in sub-Saharan Africa. AIDS. 2008;22(9):1055–61. Epub 2008/06/04. 10.1097/QAD.0b013e3282f8af84 18520349

[68] Abu-Raddad LJ, Patnaik P, Kublin JG. Dual infection with HIV and malaria fuels the spread of both diseases in sub-Saharan Africa. Science. 2006;314(5805):1603–6. Epub 2006/12/13. 10.1126/science.1132338 17158329

