# Supplementary material for: Could Circumcision of HIV-Positive Males Benefit Voluntary Medical Male Circumcision Programs in Africa? Mathematical Modeling Analysis
Source: PLoS One. 2017 Jan 24;12(1):e0170641. doi: 10.1371/journal.pone.0170641 (PMC5261810; doi:10.1371/journal.pone.0170641)
Supplement: S1 Text — (DOCX) [file pone.0170641.s001.docx]

**(I) TEXT - Supplementary Material Online**

**S1 Text**

**Further Details on the Age-Structured Mathematical (ASM) Model**

Below is a schematic illustration of the Age-Structured Mathematical (ASM) model including voluntary medical male circumcision (VMMC; S1 Fig), a narrative description of the modeled dynamics, the mathematical equations for the ASM model, and the parameters description (S1 and S2 Table). The model was programmed in MATLAB version 2015a [1]. Further description of the ASM model and parameterization can be found in Awad et al. [2].

**Narrative Description and Mathematical Equations of the ASM Model**

This model of the disease natural history and epidemiological dynamics of HIV transmission was developed with an emphasis on capturing the specific impact of VMMC by age, risk, and disease-stage described by the general states of 1) acute HIV infection, 2) latent HIV infection, and 3) advanced HIV infection. The ASM model stratifies the population into compartments according to age group, sexual risk-activity group, sex, circumcision status, and HIV status and stage of infection (S1 Fig). It consists of a set of coupled nonlinear ordinary differential equations, each of which is for a specific age and risk group.

HIV infection dynamics for females:

HIV infection dynamics for non-circumcised males:

HIV infection dynamics for circumcised males:

The population is stratified into six sexual risk groups, defined with the index $i$ ($i=1,2...6$) representing the low to higher risk groups. This stratification by risk allows the model to accommodate for the heterogeneity in sexual risk behavior in the population. The population is also stratified into 20 age groups, defined with the index $a$ ($a=1,2,...,20$), with each group representing a five-year age band (0–4, 5–9, …, 95–99 year old). The population is stratified further into three groups based on sex and male circumcision status: females, non-circumcised males, and circumcised males (subscripts$f$, $nm$, and $cm$, respectively). Definitions of all symbols in the differential equations can be found in S1 Table.

The HIV force of infection (hazard rate of infection; $\Lambda_{y}(a,i)$) experienced by each $S_{y}(a,i)$susceptible population is expressed by:

$$\Lambda_{nm}(a,i)=\rho_{S_{nm}(a,i)}\sum_{b=1}^{n_{1}} \sum_{j=1}^{n_{2}} \sum_{\alpha=1}^{3} t_{I_{\alpha,f}(b,j)\to S_{nm}(a,i)}H_{f\to m}(a,b)\mathrm{G}_{_{f\to nm}}(i,j)\frac{\rho_{I_{\alpha,f}(b,j)}I_{\alpha,f}(b,j)}{\rho_{S_{f}(b,j)}S_{f}(b,j)+\sum_{\alpha=1,2,3} \rho_{I_{\alpha,f}(b,j)}I_{\alpha,f}(b,j)}$$

$$\Lambda_{cm}(a,i)=(1-q)(1+r)\Lambda_{nm}(a,i)$$

In these expressions, $\rho_{X_{y}(a,i)}$ describes the *effective* new sexual partner change rate for each population variable $X_{y}(a,i)$ (further description below).

The parameter $r\in\left[0,\infty\right)$ models the relative increase in the effective rate of partner change due to risk compensation experienced by circumcised males following circumcision. The parameter $q\in\left[0,1\right]$ measures the efficacy of male circumcision against HIV acquisition [3-5]. At the extremes, $q=0$ implies no protection against HIV and $q=1$ implies total protection against HIV.

The HIV transmission probability per partnership between a member of the susceptible population $S_{y'}(a,i)$ and a member of the infected population $I_{\alpha,y}(a,i)$, is expressed in terms of HIV transmission probability per coital act per HIV stage in this partnership ($p_{I_{\alpha,y}(b,j)\to S_{y'}(a,i)}^{HIV}$), the frequency of coital acts per unit time in this partnership ($n_{I_{\alpha,y}(b,j)\leftrightarrow S_{y'}(a,i)}$), and the duration ($\tau_{I_{\alpha,y}(b,j)\leftrightarrow S_{y'}(a,i)}$) of this partnership:

$$t_{I_{\alpha,y}(b,j)\to S_{y'}(a,i)}=1-\left(1-p_{I_{\alpha,y}(b,j)\to S_{\gamma'}(a,i)}^{HIV}\right)^{n_{I_{\alpha,y}(b,j)\leftrightarrow S_{y'}(a,i)}\tau_{I_{\alpha,y}(b,j)\leftrightarrow S_{y'}(a,i)}}$$

The transmission probability per coital act from an HIV-positive *circumcised* male to a susceptible female is given by:

$$p_{I_{\alpha,cm}(b,j)\to S_{f}(a,i)}^{HIV}=(1-g)\times p_{I_{\alpha,nm}(b,j)\to S_{f}(a,i)}^{HIV}$$

Here, the parameter $g\in\left[0,1\right]$ is a reduction factor in the transmission probability per coital act from an HIV-positive circumcised male to a susceptible female, relative to the transmission probability per coital act from an HIV-positive non-circumcised male to a susceptible female—that is the efficacy of male circumcision against male-to-female HIV transmission. At the extremes, $g=0$ implies no protection against male-to-female HIV transmission and $g=1$ implies total protection against male-to-female HIV transmission.

The model accommodates for the mixing among the different risk groups ($G_{y\to y'}(i,j)$), and the mixing among the different age groups ($H_{y\to y'}(a,b)$ ). These two matrices provide the probability that an individual of sex $y$ in risk group $i$ and age group $a$ would choose a partner of the opposite sex $y'$ in risk group $j$ and age group $b$ [6]. The two mixing matrices are given by the expressions:

$$H_{m\to f}(a,b)=e_{1}\delta_{a,b}+\left(1-e_{1}\right)\frac{\rho_{S_{nm}(b,j)}S_{nm}(b,j)+(1+r)\rho_{S_{nm}(b,k)}S_{cm}(b,j)+\sum_{\alpha=1,2,3} \left(\rho_{I_{\alpha,nm}(b,j)}I_{\alpha,nm}(b,j)+(1+r)\rho_{I_{\alpha,nm}(b,j)}I_{\alpha,cm}(b,j)\right)}{\sum_{h=1}^{n_{2}} \left(\rho_{S_{nm}(b,h)}S_{nm}(b,h)+(1+r)\rho_{S_{nm}(b,h)}S_{cm}(b,h)+\sum_{\alpha=1,2,3} \left(\rho_{I_{\alpha,nm}(b,h)}I_{\alpha,nm}(b,h)+(1+r)\rho_{I_{\alpha,nm}(b,h)}I_{\alpha,cm}(b,h)\right)\right)}$$

$$G_{m\to f}(i,j)=e_{2}\delta_{i,j}+\left(1-e_{2}\right)\frac{\sum_{c=1}^{n_{1}} \left(\rho_{S_{nm}(c,j)}S_{nm}(c,j)+(1+r)\rho_{S_{nm}(c,j)}S_{cm}(c,j)+\sum_{\alpha=1,2,3} \left(\rho_{I_{\alpha,nm}(c,j)}I_{\alpha,nm}(c,j)+(1+r)\rho_{I_{\alpha,nm}(c,j)}I_{\alpha,cm}(c,j)\right)\right)}{\sum_{c=1}^{n_{1}} \sum_{h=1}^{n_{2}} \left(\rho_{S_{nm}(c,h)}S_{nm}(c,h)+(1+r)\rho_{S_{nm}(c,h)}S_{cm}(c,h)+\sum_{\alpha=1,2,3} \left(\rho_{I_{\alpha,nm}(c,h)}I_{\alpha,nm}(c,h)+(1+r)\rho_{I_{\alpha,nm}(c,h)}I_{\alpha,cm}(c,h)\right)\right)}$$

$$H_{f\to m}(a,b)=e'_{1}\delta_{a,b}+\left(1-e'_{1}\right)\frac{\rho_{S_{f}(b,j)}S_{f}(b,j)+\sum_{\alpha=1,2,3} \left(\rho_{I_{\alpha,f}(b,j)}I_{\alpha,f}(b,j)\right)}{\sum_{c=1}^{n_{1}} \sum_{h=1}^{n_{2}} \left(\rho_{S_{f}(c,h)}S_{f}(c,h)+\sum_{\alpha=1,2,3} \left(\rho_{I_{\alpha,f}(c,h)}I_{\alpha,f}(c,h)\right)\right)}$$

$$G_{f\to m}(i,j)=e'_{2}\delta_{i,j}+\left(1-e'_{2}\right)\frac{\sum_{c=1}^{n_{1}} \left(\rho_{S_{f}(c,j)}S_{f}(c,j)+\sum_{\alpha=1,2,3} \left(\rho_{I_{\alpha,f}(c,j)}I_{\alpha,f}(c,j)\right)\right)}{\sum_{c=1}^{n_{1}} \sum_{k=1}^{n_{2}} \left(\rho_{S_{f}(c,k)}S_{f}(c,k)+\sum_{\alpha=1,2,3} \left(\rho_{I_{\alpha,f}(c,k)}I_{\alpha,f}(c,k)\right)\right)}$$

Here, $\delta_{a,b}$ (and $\delta_{i,j}$) is the identity matrix and the parameters $e_{1}, e_{2}\text{, }e_{1}\text{'}$, and $e_{2}\text{'}\in\left[0,1\right]$ measure the degree of assortativeness in the mixing. At $e_{1}=e_{2}=e_{1}\text{'}=e_{2}\text{'}=0$, the mixing is fully proportional, while at $e_{1}=e_{2}=e_{1}\text{'}=e_{2}\text{'}=1$ the mixing is fully assortative, as individuals choose partners only from within their risk and age groups. Once the mixing matrices of one sex are determined (i.e. males), the other sex mixing matrices are determined through balance of partnerships.

**Structure of Sexual Risk Behavior**

In order to account for heterogeneity in sexual risk behavior, we incorporated six sexual risk groups in the population, starting from lower to higher levels of sexual risk behavior. It is assumed that people stay in the same risk group throughout their sexual activity lifespan (15–49 years of age).

*Distribution of the population across the different risk groups:* The proportion of the population initially in each of the six risk group was determined using a gamma distribution. The gamma distribution is motivated by the degree distribution of the number of sexual partners as identified empirically in studies in sub-Saharan Africa (SSA) [7-10]. The gamma distribution of the population across the risk groups is given by:

$$p_{y}(i)=\frac{1}{\vartheta^{k}\Gamma(k)}i^{k-1}e^{\frac{-i}{\vartheta}}.$$

Here $k$ is the shape parameter determined through normalization of the distribution, and $\vartheta$ is the scale parameter in the gamma distribution [7].

*The effective new sexual partner change rate:* Since the exact nature of sexual behavior and sexual networks in SSA is not well-understood, and varies within and across communities [11, 12], the effective new sexual partner change rate ($\rho_{X_{y}(a,i)}$) is effectively a summary measure of the population-specific level of sexual risk behavior, and captures the distribution and strength of the risk of exposure to HIV infection. The form of the $\rho_{X_{y}(a,i)}$ distribution across different risk groups was defined through a power law function. This form is motivated by simulations using an individual-based network model developed to explore the diversity in the level of sexual risk behavior [13], and also by analyses of empirical sexual networks [14], the architecture of complex weighted networks [15, 16], and the average separation between individuals in a network or a subnetwork [17, 18]. The effective new sexual partner change rate for each risk group is given by:

$\rho_{X_{y}(a,i)}=Ci^{\beta}$.

Here $C$ is a constant, determined by the average risk behavior and $\beta$ is the exponent parameter that determines the level of variability in the effective sexual partner change rate [13].

*Temporal variation in sexual risk behavior:* Given the evidence for rapidly declining HIV incidence in SSA [19-21], we incorporated temporal changes in sexual risk behavior in the model. We parameterized the temporal variation (time dependence of $\rho_{X_{y}(a,i)}$) through a Wood-Saxon function [22, 23].

$\rho_{X_{y}(a,i)}(t)=\rho_{X_{y}(a,i)}\left(1+\frac{Z}{1+\exp\left[\left(t-\xi_{Turning}\right)/\xi_{Duration}\right]}\right)$ .

This function is mathematically designed to describe and characterize transitions. It parameterizes any given transition in terms of its scale or strength, smoothness or abruptness, thickness (duration), and the turning point [22, 23]. Here, $\rho_{X_{y}(a,i)}$ is the asymptotic value of $\rho_{X_{y}(a,i)}(t)$ that describes the level of risk behavior well after the transition. $\xi_{Duration}$ describes the transition duration parameter, with the actual duration of the transition given by $\tau=4.4\xi_{Duration}$ (where the effective partner change rate falls from 90% to 10% of its initial value) [22]. Meanwhile, $\xi_{Turning}$ is the turning point year at which the effective partner change rate crosses half the way towards its asymptotic value of $\rho_{X_{y}(a,i)}$. The level of sexual risk behavior changes during the transition from $\rho_{X_{y}(a,i)}\left(1+Z\right)$ before the transition to $\rho_{X_{y}(a,i)}$ after the transition. Accordingly, the reduction in the level of sexual risk behavior is given by $l=\frac{Z}{1+Z}$.

**Increased HIV Infectiousness during Wound Healing**

The effect of increased HIV infectiousness among circumcised males who resume sexual activity before the complete healing of their circumcision wounds, was included in the model by adjusting HIV transmission probability per partnership for circumcised males through weighting transmission probability per coital act by the fraction of time spent during the duration of wound healing versus otherwise:

$$p_{I_{\alpha,cm}(b,j)\to S_{f}(a,i)}^{HIV}=\left(1-f_{resume}\right)\times p_{I_{\alpha,nm}(b,j)\to S_{f}(a,i)}^{HIV}+f_{resume}\times p_{I_{\alpha,nm}(b,j)\to S_{f}(a,i)}^{HIV}\left(\frac{R_{WH_{before}}\times d_{WH}+R_{WH_{after}}d_{I_{\beta}}}{d_{WH}+d_{I_{\beta}}}\right)$$

Transmission probability per coital act from an HIV-positive circumcised male to a susceptible female depends on the proportion of males who have sex during wound healing period ($f_{resume}\in\left[0,1\right]$), the relative risk of HIV male-to-female transmission during wound healing compared with males who are not circumcised ($R_{WH_{before}}$), the relative risk of HIV male-to-female transmission after completion of wound healing compared with males who are not circumcised ($R_{WH_{after}}$), the duration of wound healing ($d_{WH}$), and the duration circumcised males spend in each of the HIV stages of infection ($d_{I_{\beta}}=1/\omega_{I_{\beta}}-d_{WH}$).

**Parameter Values**

We parameterized our model using current empirical data on HIV epidemiology and natural history, which are listed in S2 Table along with their references. Below are some key notes/justifications on the parameters chosen based on empirical data (as opposed to model fitting):

- HIV transmission probability per coital act during each HIV stage in absence of ART ($p_{I_{Acute,i}\to S_{j}}^{HIV}=0.0107$ [acute], $p_{I_{Latent,i}\to S_{j}}^{HIV}=0.0008$ [latent], and $p_{I_{Late,i}\to S_{j}}^{HIV}=0.0042$ [advanced]), which were based on re-analyses of the Rakai Study data [24-27].
- Frequency of coital acts per month for each HIV stage ($n_{I_{Acute,j}\leftrightarrow S_{i}}=10.6$ [acute], $n_{I_{Latent,j}\leftrightarrow S_{i}}=11.0$[latent], and $n_{I_{Late,j}\leftrightarrow S_{i}}=7.1$ [advanced]), which were based on measurements of Wawer et al. [27].
- Duration of each HIV stage of infection ($1/\omega_{I_{Acute}}=49\text{ }days$ [acute], $1/\omega_{I_{Latent}}=9\text{ }years$ [latent], and $1/\omega_{I_{Acute}}=2\text{ }years$ [advanced]), which were based on data compiled by UNAIDS indicating that the average duration from HIV acquisition to death, in absence of antiretroviral therapy, is about 11 years [28, 29]. These choices were also based on Wawer et al.’s classification [27], a re-analysis of the Rakai data for acute infection [24], and the measured time from seroconversion to death in several cohort studies [19, 30].
- The rate at which people leave the population (natural mortality rate;$\mu(a)$), which was an age-dependent parameter that was determined by the country-specific average life expectancy and survival curve.
- Degree of assortativeness for the age mixing ($e_{1}=0.7$), which was fixed but with a differential age mixing where males from a specific 5-year age group will preferentially mix with females in the 5-year age group below their age group (younger females).
- Degree of assortativeness for sexual risk behavior mixing ($e_{2}=0.3$), which was a representative value based on model calibration of other epidemics in SSA [26].
- The scale parameter in the gamma distribution of the population across the different risk groups ($\vartheta=1.1$), which was based on fitting empirical data of the degree distribution (number of sexual partners over the last year year) [7].
- The exponent parameter in the power law function of the distribution of sexual risk behavior ($\beta=2.3$), which was based on analyses of sexual networks and on fitting the distribution of the clustering coefficient of all possible configurations in a sexual network [13, 14].
- The protective effect of male circumcision against HIV acquisition through female-to-male transmission ($q=60\%$), which was based on three randomized controlled trials in 2005 and 2007, demonstrating that circumcision reduces the risk of males acquiring HIV [3, 31, 32].
- Baseline (traditional) circumcision ($f_{cm}=0.13\%$), which was based on Zambia’s Demographic and Health Survey (DHS) 2007 [33].
- Risk compensation among circumcised males following VMMC (*r=*0%), which was based on the inconclusive empirical evidence to support increases in risk behavior following circumcision [34-38].
- Efficacy of VMMC against male-to-female HIV transmission (*g*) was mainly assumed to be 0%, since the evidence is mixed on this efficacy [39-46]. However, in some scenarios a *g*=20% was used based on the Weiss et al. systematic review and meta-analysis [40], and *g*=46% based on the Hallett et al. meta-analysis of two quality measures [41].
- Relative risk of HIV male-to-female transmission during wound healing compared with males not circumcised ($R_{WH_{before}}=3.5$), which was based on a clinical trial data from Uganda [39, 47].
- Relative risk of HIV male-to-female transmission after wound healing compared with males not circumcised ($R_{WH_{after}}=1$), which was based on a clinical trial data from Uganda [47].
- Proportion of males who have sex during wound healing period ($f_{resume}=24\%$) [47]
- Duration of wound healing ($d_{WH}=6\text{ }weeks$), which was based on WHO guidelines [12] and VMMC program counseling [47].
- Age-specific unit cost of VMMC, which was based on VMMC program data from Zambia [48]. The base year for US dollar is 2011. Following convention, we applied an annual discount rate of 3% on future expenditures [49].

The parameters that were determined by fitting the model to HIV prevalence time trend data are:

- Size of the epidemic in the year HIV was seeded in the simulations (1970).
- The constant $C$ parameter (in the power law function) which determines the average level of sexual risk behavior.
- The scale of the reduction in average level of sexual risk behavior in the population ($Z$).
- The duration of the sexual risk transition (the time needed for the effective partnership change rate to fall from 90% to 10% of its initial value [22, 50]; $\xi_{Duration}$).
- The turning-point year of the sexual risk behavior transition (inflexion point exactly halfway through the transition; $\xi_{Turning}$).

**References**

1. The MathWorks, Inc. MATLAB. The language of technical computing. 8.5.0.197613 (R2015a). Natick, MA, USA: ed: The MathWorks, Inc.; 2015.

2. Awad SF, Sgaier SK, Tambatamba BC, Mohamoud YA, Lau FK, Reed JB, et al. Investigating Voluntary Medical Male Circumcision Program Efficiency Gains through Subpopulation Prioritization: Insights from Application to Zambia. PLoS One. 2015;10(12):e0145729. doi: 10.1371/journal.pone.0145729. PubMed PMID: 26716442; PubMed Central PMCID: PMCPMC4696770.

3. Auvert B, Taljaard D, Lagarde E, Sobngwi-Tambekou J, Sitta R, Puren A. Randomized, controlled intervention trial of male circumcision for reduction of HIV infection risk: the ANRS 1265 Trial. PLoS Med. 2005;2(11):e298. PubMed PMID: 16231970.

4. Bailey R, editor Scaling up circumcision Programmes: The Road from Evidence to Practice. 4th IAS Conference on HIV Pathogenesis, Treatment & Prevention, July 22-25 2007, Sydney, Australia; 2007.

5. Gray RH, Li X, Kigozi G, Serwadda D, Nalugoda F, Watya S, et al. The impact of male circumcision on HIV incidence and cost per infection prevented: a stochastic simulation model from Rakai, Uganda. AIDS. 2007;21(7):845-50. PubMed PMID: 17415039.

6. Garnett GP, Anderson RM. Factors controlling the spread of HIV in heterosexual communities in developing countries: patterns of mixing between different age and sexual activity classes. Philos Trans R Soc Lond B Biol Sci. 1993;342(1300):137-59. Epub 1993/10/29. doi: 10.1098/rstb.1993.0143. PubMed PMID: 7904355.

7. Cuadros DF, Crowley PH, Augustine B, Stewart SL, Garcia-Ramos G. Effect of variable transmission rate on the dynamics of HIV in sub-Saharan Africa. BMC Infect Dis. 2011;11:216. Epub 2011/08/13. doi: 10.1186/1471-2334-11-216. PubMed PMID: 21834977; PubMed Central PMCID: PMC3175213.

8. Handcock MS, Jones JH. Likelihood-based inference for stochastic models of sexual network formation. Theor Popul Biol. 2004;65(4):413-22. Epub 2004/05/12. doi: 10.1016/j.tpb.2003.09.006. PubMed PMID: 15136015.

9. Hamilton DT, Handcock MS, Morris M. Degree distributions in sexual networks: a framework for evaluating evidence. Sex Transm Dis. 2008;35(1):30-40. Epub 2008/01/25. PubMed PMID: 18217224.

10. Bansal S, Grenfell BT, Meyers LA. When individual behaviour matters: homogeneous and network models in epidemiology. J R Soc Interface. 2007;4(16):879-91. Epub 2007/07/21. doi: 10.1098/rsif.2007.1100. PubMed PMID: 17640863; PubMed Central PMCID: PMC2394553.

11. Ferry B, Carael M, Buve A, Auvert B, Laourou M, Kanhonou L, et al. Comparison of key parameters of sexual behaviour in four African urban populations with different levels of HIV infection. AIDS. 2001;15 Suppl 4:S41-50. Epub 2001/11/01. PubMed PMID: 11686464.

12. Lagarde E, Auvert B, Carael M, Laourou M, Ferry B, Akam E, et al. Concurrent sexual partnerships and HIV prevalence in five urban communities of sub-Saharan Africa. Aids. 2001;15(7):877-84. PubMed PMID: 11399960.

13. Awad SF, Cuadros DF, Abu-Raddad LJ. Generic patterns of HIV infection distribution in human populations. Under preparation. 2012.

14. Liljeros F, Edling CR, Amaral LAN, Stanley HE, Åberg Y. The web of human sexual contacts. Promiscuous individuals are the vulnerable nodes to target in safe-sex campaigns.2001; 411.

15. Barrat A, Barthelemy M, Pastor-Satorras R, Vespignani A. The architecture of complex weighted networks. Proc Natl Acad Sci U S A. 2004;101(11):3747-52. Epub 2004/03/10. doi: 10.1073/pnas.0400087101. PubMed PMID: 15007165; PubMed Central PMCID: PMC374315.

16. Boccaletti S, Latora V, Moreno Y, Chavez M, Hwang DU. Complex networks: Structure and dynamics. Physics Reports. 2006;424(4–5):175-308. doi: 10.1016/j.physrep.2005.10.009.

17. Watts DJ, Strogatz SH. Collective dynamics of 'small-world' networks. Nature. 1998;393(6684):440-2. Epub 1998/06/12. doi: 10.1038/30918. PubMed PMID: 9623998.

18. Barabási AL. Linked: how everything is connected to everything else and what it means for business, science and everyday life: London: First Plume Printing; 2003.

19. UNAIDS/WHO. AIDS epidemic update 2010: UNAIDS fact sheet 2010. Available: <http://www.unaids.org/documents/20101123_FS_SSA_em_en.pdf>.

20. UNAIDS. UNAIDS Report on the Global AIDS Epidemic 2010 2010. Available from: <http://www.unaids.org/globalreport/Global_report.htm>.

21. Mahboob A, Haroon TS, Iqbal Z, Saleemi MA, Munir A. Prevalence of hepatitis B surface antigen carrier state in patients with lichen planus--report of 200 cases from Lahore, Pakistan. Journal of Ayub Medical College, Abbottabad : JAMC. 2007;19(4):68-70. Epub 2008/08/13. PubMed PMID: 18693602.

22. Velicia FJF. On the moments of a Wood Saxon beta distribution. Journal of Physics A: Mathematical and General. 1987.

23. Woods RD, Saxon DS. Diffuse Surface Optical Model for Nucleon-Nuclei Scattering. Physical Review. 1954;95(2):577-8. doi: Doi 10.1103/Physrev.95.577. PubMed PMID: WOS:A1954UB48500064.

24. Pinkerton SD. Probability of HIV transmission during acute infection in Rakai, Uganda. AIDS Behav. 2008;12(5):677-84. Epub 2007/12/08. doi: 10.1007/s10461-007-9329-1. PubMed PMID: 18064559.

25. Hollingsworth TD, Anderson RM, Fraser C. HIV-1 transmission, by stage of infection. J Infect Dis. 2008;198(5):687-93. Epub 2008/07/30. doi: 10.1086/590501. PubMed PMID: 18662132.

26. Abu-Raddad LJ, Longini IM, Jr. No HIV stage is dominant in driving the HIV epidemic in sub-Saharan Africa. AIDS. 2008;22(9):1055-61. Epub 2008/06/04. doi: 10.1097/QAD.0b013e3282f8af84. PubMed PMID: 18520349.

27. Wawer MJ, Gray RH, Sewankambo NK, Serwadda D, Li X, Laeyendecker O, et al. Rates of HIV-1 transmission per coital act, by stage of HIV-1 infection, in Rakai, Uganda. J Infect Dis. 2005;191(9):1403-9. Epub 2005/04/06. doi: 10.1086/429411. PubMed PMID: 15809897.

28. UNAIDS. UNAIDS Reference Group on Estimates, Modelling and Projections. 2007.

29. UNAIDS/WHO. *AIDS epidemic update 2007*.

30. UNAIDS. Epidemiological data, HIV estimates 1990-2013. 2013. Available: <http://www.unaids.org/en/dataanalysis/datatools/aidsinfo>.

31. Bailey RC, Moses S, Parker CB, Agot K, Maclean I, Krieger JN, et al. Male circumcision for HIV prevention in young men in Kisumu, Kenya: a randomised controlled trial. Lancet. 2007;369(9562):643-56. Epub 2007/02/27. doi: 10.1016/S0140-6736(07)60312-2. PubMed PMID: 17321310.

32. Gray RH, Kigozi G, Serwadda D, Makumbi F, Watya S, Nalugoda F, et al. Male circumcision for HIV prevention in men in Rakai, Uganda: a randomised trial. Lancet. 2007;369(9562):657-66. Epub 2007/02/27. doi: 10.1016/S0140-6736(07)60313-4. PubMed PMID: 17321311.

33. Zambia Demographic and Health Survey 2007. Available: <http://dhsprogram.com/pubs/pdf/FR211/FR211%5Brevised-05-12-2009%5D.pdf> [Internet]. CSO and Macro International Inc. 2009.

34. Westercamp N, Agot K, Jaoko W, Bailey RC. Risk compensation following male circumcision: results from a two-year prospective cohort study of recently circumcised and uncircumcised men in Nyanza Province, Kenya. AIDS Behav. 2014;18(9):1764-75. Epub 2014/07/23. doi: 10.1007/s10461-014-0846-4. PubMed PMID: 25047688.

35. Rosario IJ, Kasabwala K, Sadeghi-Nejad H. Circumcision as a strategy to minimize HIV transmission. Current urology reports. 2013;14(4):285-90. Epub 2013/06/19. doi: 10.1007/s11934-013-0343-8. PubMed PMID: 23775468.

36. UNAIDS. Political declaration on HIV and AIDS: Intensifying our efforts to eliminate HIV and AIDS. Geneva: UNAIDS: 2011.

37. UNAIDS. Global AIDS response progress reporting 2012: guidelines—construction of core indicators for monitoring the 2011 political declaration on HIV/AIDS. Geneva: UNAIDS: 2012.

38. UNAIDS. UNAIDS report on the global AIDS epidemic-2012. Annual UNAIDS report on the status of the AIDS epidemic and update on global initiatives to control it. Geneva: UNAIDS: 2012.

39. Wawer MJ, Makumbi F, Kigozi G, Serwadda D, Watya S, Nalugoda F, et al. Circumcision in HIV-infected men and its effect on HIV transmission to female partners in Rakai, Uganda: a randomised controlled trial. Lancet. 2009;374(9685):229-37. Epub 2009/07/21. doi: 10.1016/S0140-6736(09)60998-3. PubMed PMID: 19616720; PubMed Central PMCID: PMC2905212.

40. Weiss HA, Hankins CA, Dickson K. Male circumcision and risk of HIV infection in women: a systematic review and meta-analysis. Lancet Infect Dis. 2009;9(11):669-77. Epub 2009/10/24. doi: 10.1016/S1473-3099(09)70235-X. PubMed PMID: 19850225.

41. Hallett TB, Alsallaq RA, Baeten JM, Weiss H, Celum C, Gray R, et al. Will circumcision provide even more protection from HIV to women and men? New estimates of the population impact of circumcision interventions. Sex Transm Infect. 2011;87(2):88-93. Epub 2010/10/23. doi: 10.1136/sti.2010.043372. PubMed PMID: 20966458; PubMed Central PMCID: PMC3272710.

42. Baeten JM, Donnell D, Kapiga SH, Ronald A, John-Stewart G, Inambao M, et al. Male circumcision and risk of male-to-female HIV-1 transmission: a multinational prospective study in African HIV-1-serodiscordant couples. AIDS. 2010;24(5):737-44. Epub 2010/01/01. doi: 10.1097/QAD.0b013e32833616e0. PubMed PMID: 20042848; PubMed Central PMCID: PMC2919808.

43. Turner AN, Morrison CS, Padian NS, Kaufman JS, Salata RA, Chipato T, et al. Men's circumcision status and women's risk of HIV acquisition in Zimbabwe and Uganda. AIDS. 2007;21(13):1779-89. Epub 2007/08/11. doi: 10.1097/QAD.0b013e32827b144c. PubMed PMID: 17690577; PubMed Central PMCID: PMC2978032.

44. Gray RH, Kiwanuka N, Quinn TC, Sewankambo NK, Serwadda D, Mangen FW, et al. Male circumcision and HIV acquisition and transmission: cohort studies in Rakai, Uganda. Rakai Project Team. AIDS. 2000;14(15):2371-81. Epub 2000/11/23. PubMed PMID: 11089626.

45. Kapiga SH, Lyamuya EF, Lwihula GK, Hunter DJ. The incidence of HIV infection among women using family planning methods in Dar es Salaam, Tanzania. AIDS. 1998;12(1):75-84. Epub 1998/02/10. PubMed PMID: 9456257.

46. Kamath V, Limaye RJ. Voluntary medical male circumcision for HIV prevention and early resumption of sexual activity: a literature review. AIDS Care. 2015:1-4. doi: 10.1080/09540121.2015.1017797.

47. Hewett PC, Hallett TB, Mensch BS, Dzekedzeke K, Zimba-Tembo S, Garnett GP, et al. Sex with stitches: assessing the resumption of sexual activity during the postcircumcision wound-healing period. AIDS. 2012;26(6):749-56. doi: 10.1097/QAD.0b013e32835097ff. PubMed PMID: 22269970.

48. Vandament L. Program circumcision unit cost per actual VMMC program data from Zambia. Country-level data, Lusaka, Zambia 2013.

49. Drummond M, O’Brien B, Stoddart G, Torrance G. Methods for the economic evaluation of health care programmes. Oxford: Oxford University Press; 1999.

50. Woods RD, Saxon DS. Diffuse Surface Optical Model for Nucleon-Nuclei Scattering. Physical Review. 1954;95 (2) 577-8. doi: 10.1103/PhysRev.95.577
