# Supplementary material for: Could Circumcision of HIV-Positive Males Benefit Voluntary Medical Male Circumcision Programs in Africa? Mathematical Modeling Analysis
Source: PLoS One. 2017 Jan 24;12(1):e0170641. doi: 10.1371/journal.pone.0170641 (PMC5261810; doi:10.1371/journal.pone.0170641)
Supplement: S1 Table — (DOCX) [file pone.0170641.s002.docx]

**S1 Table. Definitions of the symbols in the equations of the age-structured mathematical (ASM) model**

| Parameter | Definition |
| --- | --- |
| $S_{y}(a,i)$ | HIV-susceptible population belonging to sex and circumcision group $y$ |
| $I_{\alpha,y}(a,i)$ | Populations with HIV infection where the index $\alpha$ marks the stage of HIV pathogenesis; $\alpha=1,2,3$ stand for acute, latent, and advanced stages, respectively |
| $N_{f}(1,i)$ | Total female population size in each sexual risk group |
| $N_{m}(1,i)$ | Total male population size in each sexual risk group |
|  | Population growth rate parameter |
| $f_{cm}$ | Fraction of the male population that is circumcised before starting sexual activity (traditional circumcision) |
| $\omega_{1}$ | Rate of progression from acute HIV infection stage to latent stage |
| $\omega_{2}$ | Rate of progression from latent HIV infection stage to late stage |
| $\omega_{3}$ | Rate of AIDS disease mortality |
| $\mu_{y}(a)$ | Natural death rate |
| $\eta(a)$ | Transition rate from one age group to the next age group |
| $\gamma(a)$ | Average rate at which non-circumcised males are being circumcised as part of a voluntary medical male circumcision intervention |
| $\Lambda_{y}(a,i)$ | HIV force of infection experienced by each $S_{y}(a,i)$susceptible population |
