# Supplementary material for: Could Circumcision of HIV-Positive Males Benefit Voluntary Medical Male Circumcision Programs in Africa? Mathematical Modeling Analysis
Source: PLoS One. 2017 Jan 24;12(1):e0170641. doi: 10.1371/journal.pone.0170641 (PMC5261810; doi:10.1371/journal.pone.0170641)
Supplement: S2 Table — (DOCX) [file pone.0170641.s003.docx]

**S2 Table.** **Model assumptions in terms of parameter values**

| Assumption | Parameter value | Sources | Range for uncertainty analysis |
| --- | --- | --- | --- |
| **HIV Biological parameters** | | |  |
| HIV transmission probability per coital act per stage of infection (**$p_{I_{\beta,i}\to S_{j}}^{HIV}$** ): | | |  |
| Acute stage | 0.0107 | [1-4] | ±20% |
| Latent stage | 0.0008 | [4, 5] | ±20% |
| Late stage  Duration of each of HIV stages ($1/\omega_{I_{\beta}}$): | 0.0042 | [1, 2, 4] | ±20% |
|  |  |  |  |
| Acute stage | 49 days | [1, 4, 6-9] | ±20% |
| Latent stage | 9.0 years | [1, 4, 6-9] | ±20% |
| Late stage | 2.0 years | [1, 4, 6-9] | ±20% |
| **Behavioral and demographic parameters** |  |  |  |
| Frequency of coital acts per HIV stage ($n_{I_{\beta,i}\leftrightarrow S_{j}}$): |  |  |  |
| Acute stage | 10.6 per month | [4] | ±20% |
| Latent stage | 11.0 per month | [4] | ±20% |
| Late stage | 7.1 per month | [4] | ±20% |
| Duration of sexual partnerships ($\tau_{I_{\beta,i}\leftrightarrow S_{j}}$): | 6 month | Representative value | ±20% |
| Degree of assortativeness for age group mixing ($e_{1}$) | 0.7 | Representative value | ±20% |
| Degree of assortativeness for risk group mixing ($e_{2}$) | 0.3 | [5] | ±20% |
| The scale parameter in the gamma distribution of the population across the risk groups ($b$) | 1.1 | [10] | ±20% |
| The exponent parameter in the power law function of the distribution of sexual risk behavior ($\alpha$) | 2.3 | [11, 12] | ±20% |
| The average level parameter of sexual risk behavior (*C*) | 0.14 | Model fitting | Model fitting |
| The scale of the reduction in average level of sexual risk behavior in the population (*Z*) | 0.59 | Model fitting | Model fitting |
| The duration of the sexual risk transition $\xi_{Duration}$ | 5.6 | Model fitting | Model fitting |
| The turning-point year of the transition $\xi_{Turning}$ | 1999 | Model fitting | Model fitting |
| **Circumcision related parameters*** |  |  |  |
| The efficacy of male circumcision against HIV acquisition among circumcised males (*q*) | 60% | [13-15] | - |
| Baseline male circumcision ($f_{cm}$) | 12.85% | [16] | - |
| The efficacy of male circumcision against male-to-female HIV transmission (*g*) | 0%  20%  46% | Representative value  [17]  [18] | - |
| Duration of wound healing ($d_{WH}$) | 6 weeks | [19] | Used in a sensitivity analysis |
| Relative risk of HIV male-to-female transmission *during* wound healing compared with males not circumcised ($R_{WH_{before}}$) | 3.5 | [19] | Used in a sensitivity analysis |
| Relative risk of HIV male-to-female transmission *after* wound healing compared with males not circumcised ($R_{WH_{after}}$) | 1 | [19] | Used in a sensitivity analysis |
| Percentage of males who have sex during wound healing period ($f_{resume}$) | 24% | [19] | Used in a sensitivity analysis |
| Age-specific unit cost of voluntary medical male circumcision (VMMC; $ USD)  0-4  5-9  10-14  15-19  20-24  25-29  30-34  35-39  40-44  45-49 | 30  90.25  90.25  95  99.75  104.74  109.97  109.97  109.97  109.97 | Based on VMMC program data [20]. | - |

* These were not included in the uncertainty analysis, as the analysis was performed to examine the impact of uncertainty in model structure parameters including HIV natural history and transmission parameters and behavioral parameters.

**References**

1. Pinkerton SD. Probability of HIV transmission during acute infection in Rakai, Uganda. AIDS Behav. 2008;12(5):677-84. Epub 2007/12/08. doi: 10.1007/s10461-007-9329-1. PubMed PMID: 18064559.

2. Hollingsworth TD, Anderson RM, Fraser C. HIV-1 transmission, by stage of infection. J Infect Dis. 2008;198(5):687-93. Epub 2008/07/30. doi: 10.1086/590501. PubMed PMID: 18662132.

3. Wawer MJ, Reynolds SJ, Serwadda D, Kigozi G, Kiwanuka N, Gray RH. Might male circumcision be more protective against HIV in the highly exposed? An immunological hypothesis. Aids. 2005;19(18):2181-2. PubMed PMID: 16284475.

4. Wawer MJ, Gray RH, Sewankambo NK, Serwadda D, Li X, Laeyendecker O, et al. Rates of HIV-1 transmission per coital act, by stage of HIV-1 infection, in Rakai, Uganda. J Infect Dis. 2005;191(9):1403-9. Epub 2005/04/06. doi: 10.1086/429411. PubMed PMID: 15809897.

5. Abu-Raddad LJ, Longini IM, Jr. No HIV stage is dominant in driving the HIV epidemic in sub-Saharan Africa. AIDS. 2008;22(9):1055-61. Epub 2008/06/04. doi: 10.1097/QAD.0b013e3282f8af84. PubMed PMID: 18520349.

6. UNAIDS. UNAIDS Reference Group on Estimates, Modelling and Projections. 2007.

7. UNAIDS/WHO. AIDS epidemic update 2007. 2007.

8. UNAIDS/WHO. AIDS epidemic update 2010: UNAIDS fact sheet 2010. Available: <http://www.unaids.org/documents/20101123_FS_SSA_em_en.pdf>.

9. UNAIDS. Epidemiological data, HIV estimates 1990-2013. 2013. Available: <http://www.unaids.org/en/dataanalysis/datatools/aidsinfo>.

10. Cuadros DF, Crowley PH, Augustine B, Stewart SL, Garcia-Ramos G. Effect of variable transmission rate on the dynamics of HIV in sub-Saharan Africa. BMC Infect Dis. 2011;11:216. Epub 2011/08/13. doi: 10.1186/1471-2334-11-216. PubMed PMID: 21834977; PubMed Central PMCID: PMC3175213.

11. Awad SF, Cuadros DF, Abu-Raddad LJ. Generic patterns of HIV infection distribution in human populations. Under preparation. 2012.

12. Liljeros F, Edling CR, Amaral LAN, Stanley HE, Åberg Y. The web of human sexual contacts. Promiscuous individuals are the vulnerable nodes to target in safe-sex campaigns.2001; 411.

13. Auvert B, Taljaard D, Lagarde E, Sobngwi-Tambekou J, Sitta R, Puren A. Randomized, controlled intervention trial of male circumcision for reduction of HIV infection risk: the ANRS 1265 Trial. PLoS Med. 2005;2(11):e298. PubMed PMID: 16231970.

14. Bailey RC, Moses S, Parker CB, Agot K, Maclean I, Krieger JN, et al. Male circumcision for HIV prevention in young men in Kisumu, Kenya: a randomised controlled trial. Lancet. 2007;369(9562):643-56. Epub 2007/02/27. doi: 10.1016/S0140-6736(07)60312-2. PubMed PMID: 17321310.

15. Gray RH, Kigozi G, Serwadda D, Makumbi F, Watya S, Nalugoda F, et al. Male circumcision for HIV prevention in men in Rakai, Uganda: a randomised trial. Lancet. 2007;369(9562):657-66. Epub 2007/02/27. doi: 10.1016/S0140-6736(07)60313-4. PubMed PMID: 17321311.

16. Zambia Demographic and Health Survey 2007. Available: <http://dhsprogram.com/pubs/pdf/FR211/FR211%5Brevised-05-12-2009%5D.pdf> [Internet]. CSO and Macro International Inc. 2009.

17. Weiss HA, Hankins CA, Dickson K. Male circumcision and risk of HIV infection in women: a systematic review and meta-analysis. Lancet Infect Dis. 2009;9(11):669-77. Epub 2009/10/24. doi: 10.1016/S1473-3099(09)70235-X. PubMed PMID: 19850225.

18. Hallett TB, Alsallaq RA, Baeten JM, Weiss H, Celum C, Gray R, et al. Will circumcision provide even more protection from HIV to women and men? New estimates of the population impact of circumcision interventions. Sex Transm Infect. 2011;87(2):88-93. Epub 2010/10/23. doi: 10.1136/sti.2010.043372. PubMed PMID: 20966458; PubMed Central PMCID: PMC3272710.

19. Hewett PC, Hallett TB, Mensch BS, Dzekedzeke K, Zimba-Tembo S, Garnett GP, et al. Sex with stitches: assessing the resumption of sexual activity during the postcircumcision wound-healing period. AIDS. 2012;26(6):749-56. doi: 10.1097/QAD.0b013e32835097ff. PubMed PMID: 22269970.

20. Vandament L. Program circumcision unit cost per actual VMMC program data from Zambia. Country-level data, Lusaka, Zambia 2013.
