# Supplementary material for: Could Circumcision of HIV-Positive Males Benefit Voluntary Medical Male Circumcision Programs in Africa? Mathematical Modeling Analysis
Source: PLoS One. 2017 Jan 24;12(1):e0170641. doi: 10.1371/journal.pone.0170641 (PMC5261810; doi:10.1371/journal.pone.0170641)
Supplement: S3 Table — (DOCX) [file pone.0170641.s004.docx]

**S3 Table. Effectiveness of the VMMC program in Zambia by 2035 under different VMMC intervention scenarios**

| **Modeled VMMC intervention scenario** | **Assumed efficacy of male circumcision against male-to-female HIV transmission** | **Effectiveness (number of VMMCs per HIV infection averted)** |
| --- | --- | --- |
| *Implications of including HIV-positive males* | | |
| A VMMC program including only HIV-negative males | 0% | 11.3 |
| Current VMMC program (less than 1% of those circumcised are HIV-positive) | 0% | 11.3 |
| A VMMC program including a proportion of HIV-positive males, based on their representation in the population | 0% | 12.5 |
| *Uptake among higher-risk males due to VMMC implementation criteria* | | |
| A VMMC program including only HIV-negative males | 0% | 11.3 |
| A VMMC program including only HIV-negative males, but with 20% lower uptake among higher-risk males | 0% | 12.7 |
| A VMMC program including a proportion of HIV-positive males, based on their representation in the population | 0% | 12.5 |
| A VMMC program including a proportion of HIV-positive males, based on their representation in the population, but with 20% higher uptake among higher-risk males | 0% | 12.2 |
| *Efficacy of male circumcision against male-to-female HIV transmission (analysis 1)* | | |
| A VMMC program including only HIV-negative males | 20% | 10.3 |
| Current VMMC program (less than 1% of those circumcised are HIV-positive) | 20% | 10.3 |
| A VMMC program including a proportion of HIV-positive males, based on their representation in the population | 20% | 10.2 |
| *Efficacy of male circumcision against male-to-female HIV transmission (analysis 2)* | | |
| A VMMC program including a proportion of HIV-positive males, based on their representation in the population | 0% | 12.5 |
| A VMMC program including a proportion of HIV-positive males, based on their representation in the population | 20% | 10.3 |
| A VMMC program including a proportion of HIV-positive males, based on their representation in the population | 46% | 8.0 |

Effectiveness is defined as the number of VMMCs needed to avert one HIV infection.

VMMC: Voluntary medical male circumcision.
