# Supplementary material for: Could Circumcision of HIV-Positive Males Benefit Voluntary Medical Male Circumcision Programs in Africa? Mathematical Modeling Analysis
Source: PLoS One. 2017 Jan 24;12(1):e0170641. doi: 10.1371/journal.pone.0170641 (PMC5261810; doi:10.1371/journal.pone.0170641)
Supplement: S1 Fig — (DOCX) [file pone.0170641.s005.docx]

**
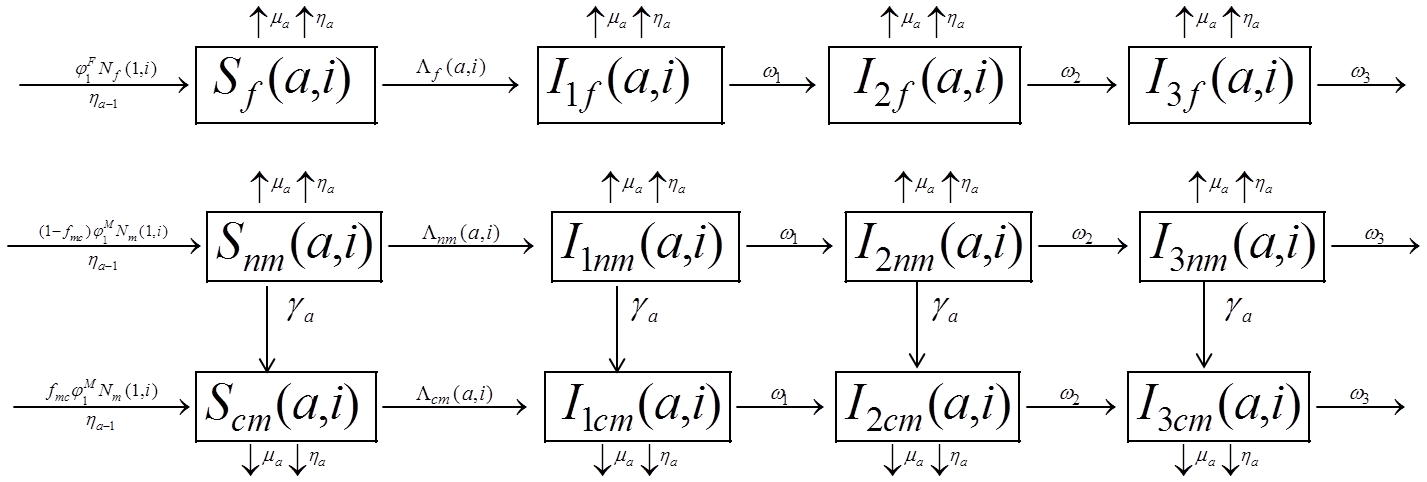
**

**S1 Fig. Schematic illustration of HIV transmission dynamics with voluntary medical male circumcision as an HIV prevention intervention.**
