# Supplementary material for: Could Circumcision of HIV-Positive Males Benefit Voluntary Medical Male Circumcision Programs in Africa? Mathematical Modeling Analysis
Source: PLoS One. 2017 Jan 24;12(1):e0170641. doi: 10.1371/journal.pone.0170641 (PMC5261810; doi:10.1371/journal.pone.0170641)
Supplement: S2 Fig — (DOCX) [file pone.0170641.s006.docx]

**S2 Fig.** **Model prediction of HIV prevalence in the 15-49 year old population in Zambia.**
