# Supplementary material for: Could Circumcision of HIV-Positive Males Benefit Voluntary Medical Male Circumcision Programs in Africa? Mathematical Modeling Analysis
Source: PLoS One. 2017 Jan 24;12(1):e0170641. doi: 10.1371/journal.pone.0170641 (PMC5261810; doi:10.1371/journal.pone.0170641)
Supplement: S3 Fig — (DOCX) [file pone.0170641.s007.docx]

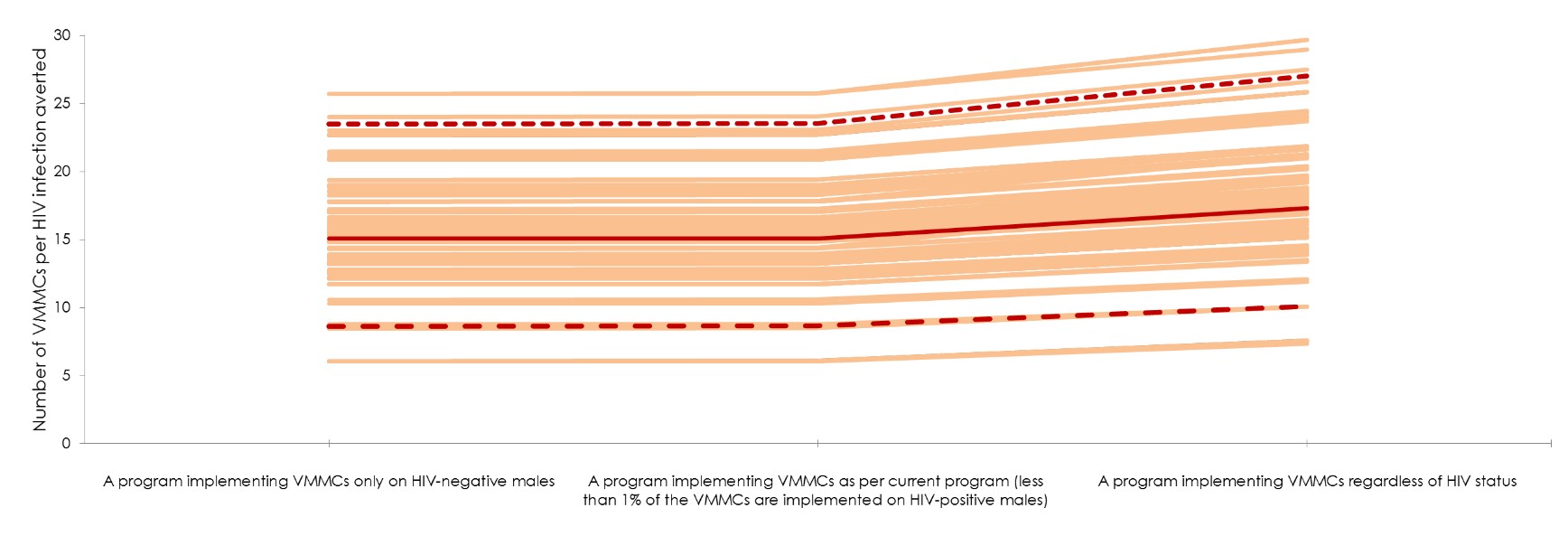


**S3 Fig. Range of uncertainty for the impact of excluding or including HIV-positive males in the VMMC program in Zambia.** The figure shows the effectiveness of the VMMC program by including *i*) only HIV-negative males, *ii*) HIV-positive males as per current program data, and *iii*) proportion of HIV-positive males, based on their representation in the population. No efficacy of male circumcision against male-to-female HIV transmission was assumed. No effect on higher-risk HIV-negative males accepting services was assumed. The solid red line represents the geometric mean of model runs, while the dashed lines bracket the 95% uncertainty interval. Effectiveness is defined as the number of VMMCs needed to avert one HIV infection.

The uncertainty analysis results should be read by following each line of each uncertainty run separately, and comparing the relative difference in VMMC effectiveness across scenarios. In all uncertainty runs, the relative difference in VMMC effectiveness was preserved indicating that there was no issue of model uncertainty about the reached conclusions for the relative effectiveness.
