# Supplementary material for: Could Circumcision of HIV-Positive Males Benefit Voluntary Medical Male Circumcision Programs in Africa? Mathematical Modeling Analysis
Source: PLoS One. 2017 Jan 24;12(1):e0170641. doi: 10.1371/journal.pone.0170641 (PMC5261810; doi:10.1371/journal.pone.0170641)
Supplement: S4 Fig — (DOCX) [file pone.0170641.s008.docx]

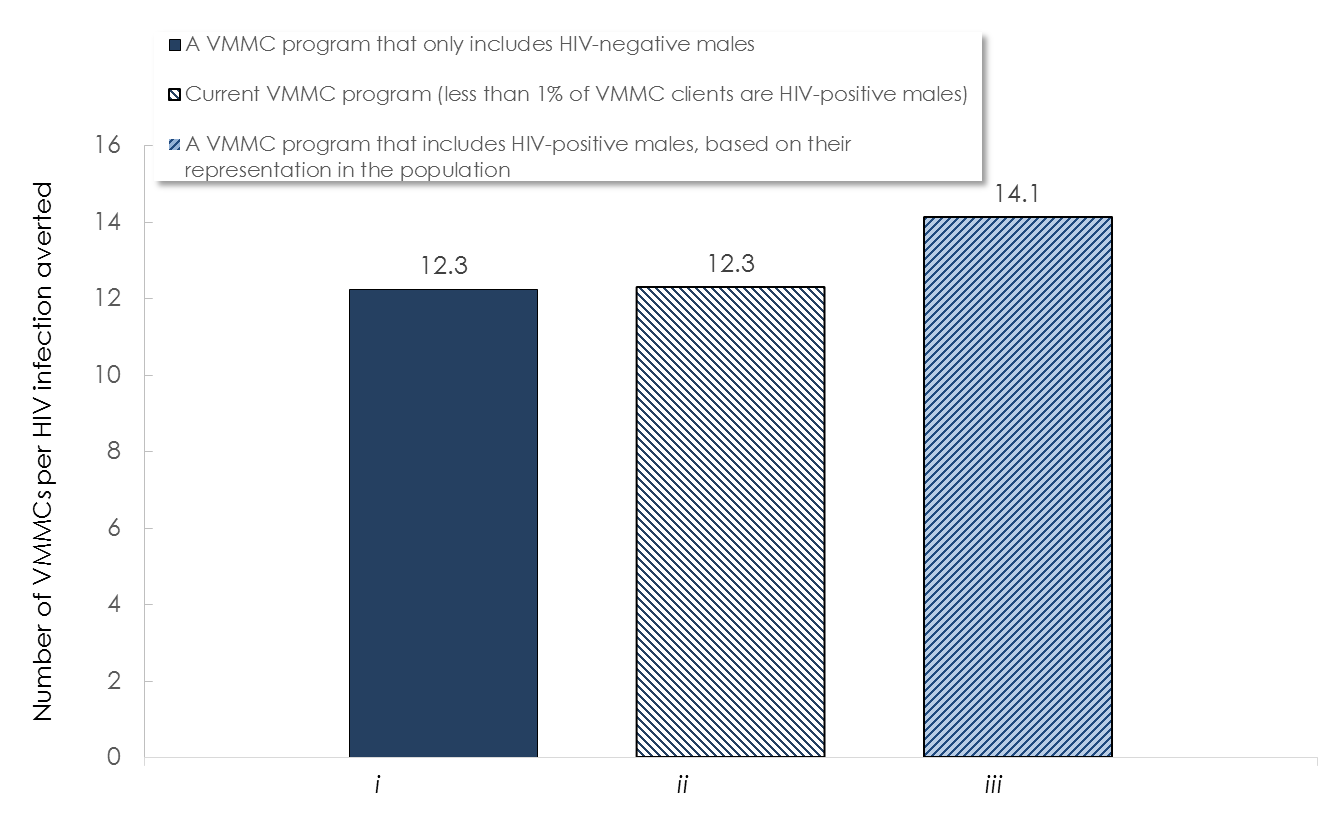


**S4 Fig. Sensitivity analysis for the impact of increased risk for male-to-female HIV transmission among circumcised males who resume their sexual activity during the healing period.** The figure shows the effectiveness of the VMMC program including *i*) only HIV-negative males, *ii*) HIV-positive males as per current program data, and *iii*) proportion of HIV-positive males based on their representation in the population. Higher HIV infectiousness among circumcised males who resume their sexual activity before the complete healing of the circumcision wound was assumed. Effectiveness is defined as the number of VMMCs needed to avert one HIV infection.

This figure reports results assuming a higher HIV infectiousness among circumcised males who resume their sexual activity before the complete healing of the circumcision wound *in all three scenarios.*
